# Lifetime Prevalence of Verbal, Physical, and Sexual Abuses in Young Elite Athletics Athletes

**DOI:** 10.3389/fspor.2021.657624

**Published:** 2021-05-31

**Authors:** Stéphane Bermon, Paolo Emilio Adami, Örjan Dahlström, Kristina Fagher, Janna Hautala, Anna Ek, Christer Anderson, Jenny Jacobsson, Carl Göran Svedin, Toomas Timpka

**Affiliations:** ^1^World Athletics, Health and Science Department, Monaco, Monaco; ^2^Laboratoire Motricité Humaine Expertise Sport Santé, Université Côte d'Azur, Nice, France; ^3^Department of Movement, Human and Health Sciences, University of Rome “Foro Italico”, Rome, Italy; ^4^Athletics Research Center, Linköping University, Linköping, Sweden; ^5^Department of Behavioural Sciences and Learning, Linköping University, Linköping, Sweden; ^6^Department of Health Sciences, Rehabilitation Medicine Research Group, Lund University, Lund, Sweden; ^7^Department of Health, Medicine, and Caring Sciences, Linköping University, Linköping, Sweden

**Keywords:** abuse, elite, harassment, junior, track and field

## Abstract

To examine prevalence of verbal, physical, and sexual abuses in young elite athletes, a cross sectional questionnaire-based survey was conducted during the World Athletics under 20 World Championships. This questionnaire aimed at distinguishing between abuses perpetrated in the context of Athletics from those which were unrelated to Athletics. Four hundred and eighty athletes (52.3%, male) from North America, South America, Europe, Africa, Asia, and Oceania took part in the electronic anonymous survey. Outside Athletics setting, no gender difference was found for the prevalence of verbal, physical, and sexual abuses. However, 45 males (18% of the male population) and 34 females (15% of the female population) athletes reported sexual abuse. Asian athletes reported a slightly higher rate of sexual abuse; three quarters of them being non-touching abuses. Inside Athletics setting, no gender difference was found for the prevalence of verbal, physical, and non-touching sexual abuses. However, 58 males (23%) and 47 females (21%) reported verbal abuses. Thirty-one males (12%) and 20 females (9%) reported physical abuses, whereas 30 males (12%) and 17 females (7%) reported sexual abuses. Physical abuses were slightly more frequent in Asia and in Africa and less frequent in South America. Sexual abuses inside Athletics also differed over regions, and were unexpectedly twice more frequent than expected in Asia and slightly less frequent than expected in Europe. Friends and partners were identified as the more frequent (>50%) abusers outside or inside the Athletics settings, whereas outside Athletics and inside Athletics, coaches were identified as sexual abuse perpetrators in 8 and 25% of cases, respectively. The prevalence of verbal, physical, or sexual abuses is high but consistent with what has been reported in United Kingdom, Norway, Canada, and Sweden at national level in recreational or elite athletes. Sexual abuse, including touching or penetrative abuses, occurred significantly more often in male athletes when compared to female athletes. This finding invites healthcare and social workers, and policymakers to also consider the risk of sexual abuse of young male athletes in Athletics. These results also call for longitudinal studies on young elite athletes.

## Introduction

Tremendous global work of preventing interpersonal violence has been achieved, but the phenomenon still exists in many sectors around the world (Burrows et al., 2018). Unfortunately, sport is not an exception, and by its global nature including both athletes from both developed and developing countries, and athletes with different socio-cultural background, gender, and age there are many forms of interpersonal violence, such as physical, verbal, and sexual abuse that the athletes could be exposed to. Moreover, athletes are often in a dependent position to for example coaches and stakeholders, which may increase the risk of being exposed to interpersonal violence. Indeed, abuses often have their starting point in a specific socio-cultural context which favors discrimination based on power differentials (Simpson et al., 2015). These power differentials are rooted in areas as diverse as sex and sexual orientation, race and ethnicity, age and disability, athletics abilities and longevity, or socio-economic status (Mountjoy et al., 2016).

Despite some recent progress in understanding the magnitude of the problem of violence against athletes in sport and the advocacy by researchers to carry out studies related to this topic, Parent and Fortier (2017) stated that this area remains under-studied within sport. When considering the amount of prevalence data available on violence in other contexts, such as child maltreatment, and school violence, there are very few studies within the sports context and especially among young elite athletes. Although a consensus statement (Mountjoy et al., 2016), or more mechanistic studies (Sundgot-Borgen et al., 2003; Timpka et al., 2014, 2019, 2020) have been published, one should acknowledge that data collection on abuses in sports are often limited to surveys performed on athletes from a single country, with a majority of studies coming from developed countries (Fasting et al., 2004; Chroni and Fasting, 2009; Alexander et al., 2011; Timpka et al., 2019). Data on elite sports is even less frequent and available.

As elite sports are organized and regulated at the international level, it is important to better describe and understand the type and magnitude of abuses on athletes from different geographical areas of the world. This is of utmost importance to help international sports governing bodies to design the most appropriate preventive and repressive actions to be implemented as an efficient athlete safeguarding policy.

There is a need for studies on abuse in elite sports including samples that are representative with regards of gender, age, and global region (Parent and Fortier, 2017) and address less studied forms of violence. Although sexual harassment and abuses in sport have been increasingly reported by athletes and media during the last years, it is important to also study the prevalence in sport of other forms of abuse such as verbal violence, which may be trivialized (Hagiwara et al., 2019; Yabe et al., 2019) or physical violence that could be perceived by the athlete as a necessary part of the training programme or the coach-athlete relationship. When studying prevalence of abuses in adolescents or young adults, the lifetime aspect is rather easy to address and is important. Indeed, the young age facilitates the recall of a possible relatively recent episodes of abuse, and their associated details and consequences. Quantitative aspects of the data collected allow the comparison between prevalence of abuses perpetrated in everyday life and abuses perpetrated in a specific sport setting, whereas the more qualitative aspects of the data can guide the authorities and policymakers on how efficiently tackling abuses in the sports settings. The specific objectives of this study were to investigate the lifetime prevalence and type of abuse experiences in a global population of adolescent elite athletics athletes, the distribution of abuse experiences by gender, global geographical region, and the environment of abuse (related or unrelated to Athletics) as well as the nature of perpetrators. We were particularly interested to know if abuse were more frequently reported:

- in some geographical area of the world,
- in female than in male elite athletes,
- in the Athletics setting when compared to everyday life,
- as being perpetrated by a coach.

## Materials and Methods

This study used a cross-sectional design. Data on verbal, physical, and sexual abuse were collected from volunteering elite athletes competing at the International Association of Athletics Federations (now World Athletics) under 20 World Championships organized in Tampere (Finland). Ethical approval was obtained from the Research Ethics Board in Linköping, Sweden (Dnr. 2018/222-31). Informed consent was obtained from all study participants or their legal representative if they were below 18 years of age. The participant could at any time drop out from the study without giving any cause. The study followed the STROBE guidelines for epidemiological research and the World Medical Association's Helsinki Principles involving research of human subjects.

### Subjects and Data Collection

The primary study population consisted of athletes who qualified and participated in the under 20 Athletics World Championships. Considering the sensitive topic of the survey, all IAAF member federations were informed of the overall objectives of the questionnaire 1 month before the Championships and asked to forward the information to their participating team 1 week before the beginning of the Championships. Upon arrival at the championships, all registered athletes received an invitation to visit the Health & Science (H&S) stands that were in the warm-up areas and team tents area. At the H&S stands, athletes had their personal accreditation badges scanned to verify their identity to ensure that they had not been scanned previously. Upon meeting these requirements, they received an invitation pamphlet with the study details and website link. They also received a once-valid unique pin-code that allowed them access to the survey page. Before starting the questionnaire, athletes were advised to be alone in a comfortable place, possibly in private so that they were not disturbed. The H&S stands were always staffed by a doctor and physiotherapist of each gender with experience of conducting studies among adolescent athletes, and support were given to athletes that needed.

### Questionnaire and Definitions

In the introduction of the questionnaire, some definitions were given to the participants. Abuse implied that a person's rights are violated by another. Physical abuse was defined as “deliberately hurting a person causing injuries such as bruises, broken bones, burns, or cuts” (Jernbro and Janson, 2018). Sexual abuse was defined as “any sexual interaction with person(s) of any age that is perpetrated against the victim's will, without consent or in an aggressive, exploitative, manipulative, or threatening manner.”

A web questionnaire was developed by researchers with experience in sports medicine, social medicine, and psychiatry who were all experienced in designing and running questionnaires in sports and social medicine. The questionnaire was then translated in the following languages: English, French, Spanish, Portuguese, Japanese, Chinese, Arabic, and Amharic. The questionnaire data were stored live in a secured cloud platform (Briteback®, Norrkoping, Sweden). The questionnaire first asked for demographics (age, sex, geographical area of origin) and Athletics background (age when started Athletics, type of Athletics event, training volume).

Second, lifetime verbal, physical, and sexual abuses within or outside Athletics settings were asked for (see full questionnaire in eight languages in the Supplementary Material). The questionnaire asked whether it had happened that an adult had done any of the following to the athlete: “(a) Pushed, shoved or shook you up, (b) Thrown something at you, (c) Hurt you with her/his hands, (d) Kicked, bit or hit you with her/his fists, (e) Hurt you with a weapon, (f) Burned or scalded you, (g) Tried to smother you (took stranglehold), and (h) Physically attacked you otherwise.” Precisely, the variable used to collect data on sexual abuse was derived from the statement and questions originally developed by Mossige (2001) and used in earlier Swedish studies (Timpka et al., 2019): “Sometimes people are persuaded, pressed, or forced to do sexual activities they cannot protect themselves from. The following questions are about such situations. Have you been exposed to any of the following against your will?” (it is possible to choose several alternatives): (a) “Somebody exposed himself or herself indecently toward you, (b) Somebody has pawed (touched your body in an indecent way) you, (c) You masturbated somebody else, (d) You have had sexual intercourse, (e) You have had oral sex, and (f) You have had anal sex.” To assess verbal abuse, the questionnaire asked whether it happened that someone had done any of the following to the athlete: “(a) Insulted you, (b) Threatened to hit you, (c) Isolated you from friends.”

Before implementing the questionnaire, several pilot tests within the research group were conducted, using either the English or French versions of the questionnaire. These pilot tests confirmed that young adults understood the purpose of the study as well as the questions and items contained in the questionnaire. A similar version of this questionnaire had already been validated and used in a previous study on a similar target population (Timpka et al., 2014, 2019).

### Data Analysis

To examine patterns of drop-outs non-participation per sex and per geographical area were first analyzed using chi-squared tests. The data on lifetime verbal, physical, and sexual abuse were categorized by gender, setting where the abuse was perpetrated and geographical area (North America, South America, Europe, Africa, Asia, Oceania). Athletics events were recoded into jumps, throws, sprints, combined events, middle- and long-distance running, and race walking. We thereafter performed descriptive statistics on demographics, verbal, physical, and sexual abuses. Prevalence is calculated and expressed as absolute number of observations, percentage, and 95% confidence interval (see Supplementary Materials). Differences between frequencies are tested with a χ^2^-test, association between variables is tested with the Cramér's V-test, and effect size are considered as small, medium, and large for V-value above 0.1, 0.3, and 0.5, respectively. Statistical analysis was conducted in R (R Core Team, 2020).

## Results

Following an internal check for validity and completeness, a total of 480 individual questionnaires were used to perform the statistical analysis. With 1,322 athletes taking part in the Championships this represents a response rate of 36.3%. There were no differences in gender-proportion between eligible and participating athletes [χ^2^(1, *n* = 1,322) = 0.05, Cramér's V = 0.006]. The proportion of athletes participating in the study varied from one geographical area to another [χ^2^(5, *n* = 1,322) = 35.32, Cramer's V = 0.16], showing higher than expected participation in South American and Asian athletes and lower participation in their North American peers (Table 1). Distribution of study participants' age and their age when started Athletics is shown in Table 2. There was no observable age difference in the age subgroups (below 18 years old; 18–20 years old) between male and female athletes who took part to the study [χ^2^(1, *n* = 456) = 2.93, Cramér's V = 0.08]. Sprinters and middle- and long-distance runners together represented more than 60% of the studied population (Table 3). Female athletes below and above 19 years old trained 14.6 h (standard deviation: 5.6 h) and 15.4 h (6.5 h) per week, respectively. Male athletes below and above 19 years old trained 14.3 h (6.1 h) and 15.8 h (6.2 h) per week, respectively.

**Table 1:** Proportion of athletes participating to the study, displayed by sex, and geographical area.

	**Athletes participating to the survey/Athletes accredited to the World Championships and %**													
	**North America**		**South America**		**Europe**		**Africa**		**Asia**		**Oceania**		**Total**	
Male	35/128	27.3%	16/27	59.3%	126/308	40.9%	28/99	28.3%	36/101	35.6%	10/35	28.6%	251	36.0%
Female	35/131	26.7%	28/36	**77.8%**	104/310	33.5%	9/38	23.7%	44/73	**60.3%**	9/36	25.0%	229	36.7%
Total	70/259	27.0%	44/63	**69.8%**	230/618	37.2%	37/137	27.0%	80/174	**46.0%**	19/71	26.8%	480	36.3%

***Bold** values indicates unexpectedly high participation proportions and underlined values indicates unexpectedly low participation proportions*.

**Table 2:** Participants' age and athletics history by gender and geographical area.

	**North America**			**South America**			**Europe**			**Africa**			**Asia**			**Oceania**			**World**		
	**F**	**M**	**All**	**F**	**M**	**All**	**F**	**M**	**All**	**F**	**M**	**All**	**F**	**M**	**All**	**F**	**M**	**All**	**F**	**M**	**All**
**Age**																					
<19 years	21	18	39	22	9	31	69	76	145	7	15	22	24	18	42	6	7	13	149	143	292
	64%	55%	59%	81%	64%	76%	68%	64%	66%	88%	56%	63%	56%	53%	55%	86%	70%	76%	68%	60%	64%
19–20 years	12	15	27	5	5	10	32	43	75	1	12	13	19	16	35	1	2	4	70	94	164
	36%	45%	41%	19%	36%	24%	32%	36%	34%	12%	44%	37%	44%	47%	45%	14%	30%	24%	32%	40%	36%
**Age when starting athletics**																					
<8 years	10	9	19	2	5	7	28	39	67	3	5	8	8	4	12	1	2	3	52	64	116
	29%	26%	27%	7%	31%	16%	27%	31%	29%	33%	19%	22%	18%	11%	15%	11%	20%	16%	23%	26%	24%
8–12 years	11	12	23	12	4	16	40	34	74	3	6	9	24	11	35	7	3	10	97	70	167
	31%	34%	33%	43%	25%	36%	38%	27%	32%	33%	22%	25%	55%	31%	44%	78%	30%	53%	42%	28%	35%
>12 years	14	14	28	14	7	21	36	53	89	3	16	19	12	20	32	1	5	6	80	115	195
	40%	40%	40%	50%	44%	48%	35%	42%	39%	33%	59%	53%	27%	57%	41%	11%	50%	32%	35%	46%	41%

*Results are presented as absolute numbers and percentage of total per gender and geographical area. M, Male; F, Female*.

**Table 3:** Distribution of athletes by geographical area, gender, and event/discipline.

	**North America**			**South America**			**Europe**			**Africa**			**Asia**			**Oceania**			**World**		
	**F**	**M**	**All**	**F**	**M**	**All**	**F**	**M**	**All**	**F**	**M**	**All**	**F**	**M**	**All**	**F**	**M**	**All**	**F**	**M**	**All**
Jumps	4	2	6	3	6	9	16	18	34	0	1	1	7	7	14	0	0	0	30	34	64
	11%	6%	9%	11%	38%	20%	15%	14%	15%	0%	4%	3%	16%	19%	18%	0%	0%	0%	13%	14%	13%
Throws	3	6	9	5	0	5	26	19	45	2	1	3	5	2	7	2	1	3	43	29	72
	9%	17%	13%	18%	0%	11%	25%	15%	20%	22%	4%	8%	11%	6%	9%	22%	10%	16%	19%	12%	15%
Sprints	11	11	22	11	6	17	29	59	88	4	16	20	14	16	30	3	2	5	72	110	182
	31%	31%	31%	39%	38%	39%	28%	47%	38%	44%	57%	54%	32%	44%	38%	33%	20%	26%	31%	44%	38%
MLD running	12	10	22	1	3	4	22	24	46	3	10	13	15	7	22	3	5	8	56	59	115
	34%	29%	31%	4%	19%	9%	21%	19%	20%	33%	36%	35%	34%	19%	28%	33%	50%	42%	24%	24%	24%
Combined events	4	0	4	0	0	0	8	2	10	0	0	0	1	0	1	0	2	2	13	4	17
	11%	0%	6%	0%	0%	0%	8%	2%	4%	0%	0%	0%	2%	0%	1%	0%	20%	11%	6%	2%	4%
Race walk	1	6	7	8	1	9	3	4	7	0	0	0	2	4	6	1	0	1	15	15	30
	3%	17%	10%	29%	6%	20%	3%	3%	3%	0%	0%	0%	5%	11%	8%	11%	0%	5%	7%	6%	6%
Total	35	35	70	28	16	44	104	126	230	9	28	37	44	36	80	9	10	19	229	251	480
	100%	100%	100%	100%	100%	100%	100%	100%	100%	100%	100%	100%	100%	100%	100%	100%	100%	100%	100%	100%	100%

*Results are presented as absolute numbers and percentage of total per gender and geographical area. M, Male; F, Female; MLD, middle- and long-distance running*.

Absolute numbers and proportions of lifetime verbal, physical, and sexual abuses reported inside an outside the Athletics setting are presented in Table 4.

**Table 4:** Number and proportions of lifetime verbal, physical, and sexual abuses (inside an outside the Athletics setting) victims in the study population displayed by gender and geographical area (*n* = 480).

	**North America**		**South America**		**Europe**		**Africa**		**Asia**		**Oceania**		**Total**	
**OUTSIDE ATHLETICS**														
**Male**														
Verbal abuses	7	20%	4	25%	35	28%	8	29%	16	44%	1	10%	71	28%
Physical abuses	2	6%	3	19%	20	16%	5	18%	10	28%	1	10%	41	16%
Sexual abuses	2	6%	5	31%	21	17%	6	21%	11	31%	0	0%	45	18%
**Female**														
Verbal abuses	13	37%	4	14%	27	26%	4	44%	9	20%	1	11%	58	25%
Physical abuses	7	20%	1	4%	20	19%	3	33%	10	23%	1	11%	42	18%
Sexual abuses	4	11%	0	0%	15	14%	2	22%	12	27%	1	11%	34	15%
**INSIDE ATHLETICS**														
**Male**														
Verbal abuses	4	11%	3	19%	29	23%	8	29%	14	39%	0	0%	58	23%
Physical abuses	2	6%	0	0%	14	11%	5	8%	10	28%	0	0%	31	12%
Sexual abuses	1	3%	3	19%	14	11%	4	14%	8	22%	0	0%	30	12%
**Female**														
Verbal abuses	12	34%	3	11%	18	17%	4	44%	9	20%	1	11%	47	21%
Physical abuses	4	11%	0	0%	6	6%	3	33%	7	16%	0	0%	20	9%
Sexual abuses	5	14%	0	0%	2	2%	1	11%	8	18%	1	11%	17	7%

*Results are expressed as absolute and relative values. Relative values are calculated as percentages of the total number of subjects per gender and geographical area*.

### Abuses Perpetrated Outside the Athletics Setting

There was no difference between genders for verbal, physical, and sexual abuses [χ^2^(1, *n* = 480) = 0.533, Cramér's V = 0.03]; [χ^2^(1, *n* = 480) = 0.337, Cramér's V = 0.03]; [χ^2^(1, *n* = 480) = 0.827, Cramér's V = 0.04]; respectively. There was no difference between geographical areas for verbal and physical abuses outside Athletics. There were, however, small differences in experience of sexual abuse outside Athletics over the regions [χ^2^(5, *n* = 480) = 14.00, Cramer's V = 0.17] and sexual abuse was more frequently reported from Asian athletes. A closer look at characteristics of these sexual abuses reported by young Asian athletes showed that at least three quarters of them were non-touching sexual abuses such as sexual poses or exposing of genitals (Table 5). Mean age for first incident of verbal, physical, or sexual abuses outside Athletics are presented in Table 6. Among the recorded sexual abuses perpetrated outside the Athletics setting female athletes reported 15 cases of touching sexual abuses. According to our questionnaire, touching sexual abuses means the perpetrator touched the genitals or tried to undress, forced for perpetrator's masturbation, vaginal intercourse, oral sex, or anal sex. Male athletes reported 24 cases of touching sexual abuses unrelated to the sport of Athletics. Type of sexual abuse perpetrated outside Athletics displayed by global geographical area are presented in Supplementary Material (Table 9). More than half of sexual abuses involved friends and partners (see Table 7).

**Table 5:** Lifetime sexual abuse experiences outside Athletics displayed by global geographical region.

	**North America**	**South America**	**Europe**	**Africa**	**Asia**	**Oceania**	**Total**
**Females**							
No-touching sexual abuse	2 (6%)	0 (0%)	7 (7%)	1 (11%)	10 (23%)	1 (11%)	21 (9%)
Touching sexual abuse	2 (6%)	0 (0%)	9 (9%)	1 (11%)	3 (7%)	0 (0%)	15 (7%)
Any sexual abuse	4 (11%)	0 (0%)	15 (14%)	2 (22%)	12 (27%)	1 (11%)	34 (15%)
*n* (%)	35 (100%)	28 (100%)	104 (100%)	9 (100%)	44 (100%)	9 (100%)	229 (100%)
**Males**							
No-touching sexual abuse	0 (0%)	2 (12%)	12 (10%)	4 (14%)	8 (22%)	0 (0%)	26 (10%)
Touching sexual abuse	2 (6%)	4 (25%)	12 (10%)	3 (11%)	3 (8%)	0 (0%)	24 (10%)
Any sexual abuse	2 (6%)	5 (31%)	21 (17%)	6 (21%)	11 (31%)	0 (0%)	45 (18%)
*n* (%)	35 (100%)	16 (100%)	126 (100%)	28 (100%)	36 (100%)	10 (100%)	251 (100%)
**All**							
No-touching sexual abuse	2 (3%)	2 (5%)	19 (8%)	5 (14%)	18 (22%)	1 (5%)	47 (10%)
Touching sexual abuse	4 (6%)	4 (9%)	21 (9%)	4 (11%)	6 (8%)	0 (0%)	39 (8%)
Any sexual abuse	6 (9%)	5 (11%)	36 (16%)	8 (22%)	23 (29%)	1 (5%)	79 (16%)
*n* (%)	70 (100%)	44 (100%)	230 (100%)	37 (100%)	80 (100%)	19 (100%)	480 (100%)

**Table 6:** Mean age for first incident of verbal, physical, or sexual abuses outside and inside the Athletics setting.

	**Outside Athletics (year)**	**Inside Athletics (year)**
Verbal and/or physical abuse	13.6 (12.5–14.7) *n* = 45	14.7 (13.2–16.2) *n* = 27
Sexual abuse	14.4 (11.4–17.3) *n* = 11	15.7 (12.9–18.5) *n* = 3
Verbal and/or physical abuse	13.4 (12.1–14.6) *n* = 35	15.4 (14.5–16.3) *n* = 25
Sexual abuse	14.9 (12.8–17) *n* = 16	13.6 (10.1–17.1) *n* = 7

*Results are expressed as mean (95% confidence interval)*.

**Table 7:** Type and distribution of first abuse perpetrators outside and inside the Athletics setting.

**Perpetrators**	**Outside Athletics**	**Inside Athletics**
	**(*n* = 35) (%)**	**(*n* = 12) (%)**
Parent	3	0
Sibling	3	0
Relative	3	8
Friend	29	42
Partner	29	8
Athlete	8	8
Trainer or Coach	8	25
Manager	0	0
Teacher	3	0
Unknown	14	9

*When mentioned outside the Athletics setting, athlete, trainer, or coach are considered as perpetrators unrelated to Athletics*.

### Abuses Perpetrated Inside the Athletics Setting

There was no difference between genders for verbal, physical, and sexual abuses [χ^2^(1, *n* = 480) = 0.468, Cramér's V = 0.03]; [χ^2^(1, *n* = 480) = 1.65, Cramér's V = 0.06]; [χ^2^(1, *n* = 480) = 2.78, Cramér's V = 0.08]; respectively. There was no difference between geographical areas for verbal abuses inside Athletics [χ^2^(5, *n* = 480) = 9.80, Cramér's V = 0.14]. Physical abuses in the Athletics setting differed over the regions (Cramer's V = 0.22), and were twice more frequent than expected in Asia and Africa and less frequent than expected in South America. Sexual abuses inside Athletics also differed over regions, [χ^2^(5, *n* = 480) = 16.00, Cramer's V = 0.18] and were twice more frequent than expected in Asia and slightly less frequent than expected in Europe. Mean age for first incident of verbal, physical, or sexual abuses inside Athletics are presented in Table 6. Touching sexual abuses in an athletics setting was more reported by male (17 observations) than female (6 observations) athletes [χ^2^(1, *n* = 480) = 4.526, Cramér's V = 0.10; Table 8]. Type of sexual abuse perpetrated inside Athletics displayed by global geographical area are presented in Supplementary Material (Table 10). Friends and athletics coaches together represented two-thirds of the perpetrators of sexual abuses (Table 7).

**Table 8:** Lifetime sexual abuse experiences inside Athletics displayed by global geographical region.

**Sexual abuse**	**North America**	**South America**	**Europe**	**Africa**	**Asia**	**Oceania**	**Total**
**Females**							
No-touching sexual abuse	2 (6%)	0 (0%)	1 (1%)	1 (11%)	8 (18%)	0 (0%)	12 (5%)
Touching sexual abuse	4 (11%)	0 (0%)	1 (1%)	0 (0%)	0 (0%)	1 (11%)	6 (3%)
Any sexual abuse	5 (14%)	0 (0%)	2 (2%)	1 (11%)	8 (18%)	1 (11%)	17 (7%)
*n* (%)	35 (100%)	28 (100%)	104 (100%)	9 (100%)	44 (100%)	9 (100%)	229 (100%)
**Males**							
No-touching sexual abuse	1 (3%)	2 (12%)	4 (3%)	2 (7%)	5 (14%)	0 (0%)	14 (6%)
Touching sexual abuse	0 (0%)	2 (12%)	10 (8%)	2 (7%)	3 (8%)	0 (0%)	17 (7%)
Any sexual abuse	1 (3%)	3 (19%)	14 (11%)	4 (14%)	8 (22%)	0 (0%)	30 (12%)
*n* (%)	35 (100%)	16 (100%)	126 (100%)	28 (100%)	36 (100%)	10 (100%)	251 (100%)
**All**							
No-touching sexual abuse	3 (4%)	2 (5%)	5 (2%)	3 (8%)	13 (16%)	0 (0%)	26 (5%)
Touching sexual abuse	4 (6%)	2 (5%)	11 (5%)	2 (5%)	3 (4%)	1 (5%)	23 (5%)
Any sexual abuse	6 (9%)	3 (7%)	16 (7%)	5 (14%)	16 (20%)	1 (5%)	47 (10%)
*n* (%)	70 (100%)	44 (100%)	230 (100%)	37 (100%)	80 (100%)	19 (100%)	480 (100%)

## Discussion

The specific objectives of this study were to investigate the lifetime prevalence and type of abuse experiences in a global population of adolescent elite athletics athletes, the distribution of abuse experiences by gender, global geographical region, and the environment of abuse (related or unrelated to Athletics) as well as the nature of perpetrators.

### Prevalence in Different Geographical Areas

To the best of our knowledge, this study is the first to report lifetime prevalence of abuse experiences in a global population of adolescent and young elite athletes. World Athletics, with its 214 member federations, represents a global sport where occurrence, perception, and report of abuses may vary because of different, cultural, religious, and economical backgrounds. Therefore, knowledge of these variations is of the essence when prevention programs are prepared. The high prevalence of sexual abuse perpetrated inside and outside the Athletic setting, in Asia, is an unexpected finding. In our study, the Asian geographical area consistently demonstrated the highest reported rates of verbal and physical abuses. Such findings are likely to be attributed to strong differences between Asian and Western countries in child rearing. Indeed, and as noted by Wu (1981), the most “*abusive*” Asian parent is one who does not properly discipline his or her child, and consequently, “*drowns the child with love*.” As a result, Asian parents, because of a collectivist, rather than an individualist value orientation, teach their children to view their role within the family and society in terms of relationships and obligations. Meston et al. (1999), investigating self-report of abuse among undergraduate students of different backgrounds, have also shown that in addition to reporting a higher incidence of physical and emotional abuse, Asians were more likely than their non-Asian counterparts to perceive themselves as having been physically and emotionally abused. Our actual data confirm these findings, emphasizing on the importance of cultural and educational contexts when designing and running studies on abuse involving different ethnical groups.

### Prevalence of Verbal and Physical Abuses

Verbal abuse is often trivialized in many societies, especially in younger population. Adolescents and young adults who communicate a lot through social media, increase their risks of mood and anxiety disorders through cyberbullying; a modern form of verbal abuse (O'Reilly et al., 2018). It is also considered by some as a minor form of abuse and therefore, poorly reported by the victims and likely underestimated in official reports. Among our young elite athletes, this type of abuse is frequent and reported by 27 and 22% of them in non-athletics and athletics settings, respectively. As this kind of abuse may occur more frequently than physical and sexual abuses, its negative consequence on health and motivation for sport (Yabe et al., 2019) should not be underestimated.

Eleven percent of our young elite athlete population reported physical abuse occurring in connection with Athletics. This is lower than the 14.9% reported in junior Swedish athletes (Timpka et al., 2019), and the 24% in United Kingdom sports teenagers (Alexander et al., 2011). Like these authors we observed a slightly higher rate of physical abuse in males (12%) in comparison to females (9%). Outside the Athletics setting, 17.5% of our elite population reported physical abuse. Although the observed prevalence of verbal and physical abuses in connection with athletics are lower than in everyday life, it is worrying that competitive sport does not really protect against this type of abuse.

### Prevalence of Sexual Abuse and Description of the Perpetrators

In comparison, one out of six and one out of ten young elite athletes experienced sexual abuse unrelated and related to Athletics, respectively. These numbers are high but consistent with some other reports. Parent et al. (2016) found a 10.2% rate of lifetime sport-related sexual abuse among 14–17-year-old adolescent from the province of Quebec. Using a screening questionnaire of 2,118 Australian athletes, Leahy (2001) found that 12% of the female athletes reported experiencing sexual abuse within the sport environment at some time in their lives. For female and male athletes, the mean age for the first incident of sexual abuse associated with Athletics was 13.2 and 15.7 years, respectively. Epidemiological data on age of victims when experienced their first sexual abuse does not exist at international sport level. However, the mean age we report here for female athletes is quite young and show that some young female athletes have been abused for the first time only a couple of years after attending regular participation in athletics.

In our study, the higher prevalence of sexual abuses in male athletes compared to female athletes is an unexpected result. This phenomenon exists inside and outside the Athletics setting and is noteworthy in four different geographical areas. A more detailed analysis also revealed that touching sexual abuses were more frequent in males than in females, both in absolute and relative numbers. This represented 35% of all sexual abuses in women and 57% in men. Moreover approximately 34% of sexual abuses experienced by male athletes are penetrative abuses, whereas this proportion varies from 10% (outside Athletics) to 14% (inside Athletics) in female athletes. Although previous research positioned females as the most frequent victims of sexual abuse, our predominantly male count of victims of sexual abuse is not entirely new. Indeed, Alexander et al. (2011), studying a large cohort of children and teenagers participating in organized sports in the United Kingdom, reported sexual harm in 5% of male and 2% of female population. Our results confirm that sexual abuse in young male athlete is important and probably underestimated (Hartill, 2009; Parent and Bannon, 2012). Further research and surveys are needed to better characterize the sexual abuses against boys and understand if this finding is specific to this age group, and the sport context.

In our study, a group consisting of friends, athletes, and partners represented 58% of the perpetrators of abuse in athletics whereas coaches and trainers represented 25%. Our description is consistent with what has been reported in the few similar studies. Indeed, Alexander et al. (2011) found that teammates and peers were responsible for 70% of sexual harassment and 80% of sexual harm in organized sports, whereas coaches were involved in 15 and 11%, respectively. This is confirmed by Vertommen et al. (2016) who retrospectively studied 1,785 adults with interpersonal violence in sport before the age of 18 and showed that peers are the perpetrators in 45% of cases of sexual violence. This data confirms that, contrary to common belief, athletics coaches, and trainers are not the main perpetrators of sexual abuses in young elite Athletic athletes. However, one should note a coach career lasts several decades during which successive generations of athletes can be abused by a single person (Toftegaard Nielsen, 2001). Moreover, Vertommen et al. (2016) revealed from a survey conducted in Belgium and the Netherland that acts of sexual violence committed by coaches are significantly more severe in comparison to acts committed by peer athletes and other perpetrators. Our study identified friends as the abusers in 42% of sexual abuses occurring in connection with Athletics. As alternative answers to this question such as partner or athletes were less chosen, this suggests that individuals who were not involved in any aspects of Athletics were likely perpetrators of sexual abuse around athletics training facilities and stadium.

### Study Strengths and Limitations

The elite and the international nature of the sample studied is unique, but one should remain cautious not to extrapolate this study findings and conclusions to sub-elite, recreational, or older athletes. Background variables such as well-being scores (Topp et al., 2015), injury (sports- and non-sports-related) rate during the previous 12 months, information on how victims felt and coped following experiences of abuse have been collected (see full questionnaire in English and French in the Supplementary Material), and used to perform within-person and multivariate analyses whose results will be presented in another manuscript. Our questionnaire poorly addressed some forms of abuse such as neglect which is a psychological abuse with potential physical consequences (Mountjoy et al., 2016). Because of the very diverse geographical origin, and access to internet and social media in our studied population, we had decided not to develop the cyber-bullying aspect of our questionnaire. However, we recommend that his form of verbal abuse which prevalence is increasing with the development of social media (O'Reilly et al., 2018), is included in future studies on abuse in sports. From a study survey standpoint, we have noted, while analyzing the results, that the first part of the survey (dealing with verbal and physical abuse) asks about victimization from an adult perpetrator whereas the second part (dealing with sexual abuse) asks about any kind of perpetrators. This could have caused an information bias with some respondents misinterpreting both parts of the questionnaire as pertaining to adult perpetrators only. The English version of the questionnaire contains an ambiguous translation regarding masturbation as a sexual abuse (questions 20 and 23). After checking the other translations, we confirm that it was the perpetrator's genitals that were involved (see Supplementary Materials). Our study design did not allow to measure the recurrence of sexual abuse in the athletics setting with times, and the contribution of coaches to this misconduct.

## Conclusions

In our studied population of young elite Athletics athlete, prevalence of verbal, physical, or sexual abuse is high but globally consistent with what has been reported in elite athletes in countries like Australia, Canada, or Sweden. Higher rates of abuses are found in some geographical areas, compared to others, but it is important to first define the nature of the abuse accurately, and then to contextualize the results with (i) the respective cultural backgrounds, (ii) the very specific nature of the Athletics population we have studied, before drawing any definitive conclusions. These geographical trends should be confirmed or refuted by further studies involving international elite athletes. Lifetime experiences of verbal and physical abuse among young elite athletes inside or outside the Athletics must not be neglected as they are disturbingly prevalent and confirm that the competitive Athletics setting is not a place immune to all forms of abuse. Prevalence of verbal and physical abuses inside and outside the Athletics setting are similar between young male and female elite athletes. However, as already reported by other authors, touching sexual abuse occurs more often in male athletes when compared to female athletes. This difference persists when the more severe forms of penetrative sexual abuses are considered. The mechanisms underlying these sexual abuses, their possible recurrence, and their consequences for mental health and injury rates and severity need to be further investigated through multivariate analysis and longitudinal studies. Moreover, the methods used in this study should be replicated at the club level to measure prevalence of verbal, physical, and sexual abuses in Athletics at sub-elite and recreational levels. The results obtained from existing and future studies will improve our understanding of the mechanisms and consequences of abuses and help healthcare and social workers, policymakers to design and implement repressive actions as well as primary and secondary prevention campaigns against verbal, physical, and sexual abuses.

## Data Availability Statement

The raw data supporting the conclusions of this article will be made available by the authors, without undue reservation.

## Ethics Statement

The studies involving human participants were reviewed and approved by Research Ethics Board in Linköping, Sweden (Dnr. 2018/222-31). Written informed consent to participate in this study was provided by the participants' legal guardian/next of kin.

## Author Contributions

All authors listed have made a substantial, direct and intellectual contribution to the work, and approved it for publication.

## Conflict of Interest

The authors declare that the research was conducted in the absence of any commercial or financial relationships that could be construed as a potential conflict of interest.
