# Supplementary material for: Lifetime Prevalence of Verbal, Physical, and Sexual Abuses in Young Elite Athletics Athletes
Source: Front Sports Act Living. 2021 May 31;3:657624. doi: 10.3389/fspor.2021.657624 (PMC8200562; doi:10.3389/fspor.2021.657624)
Supplement: Supplementary file 1 [file Table_1.DOCX]

**Table 1.** Confidence intervals for prevalence of lifetime verbal, physical and sexual abuses (inside an outside the Athletics setting) victims in the study population displayed by gender, and geographical area (n=480).

|  | North America | | South America | | Europe | | Africa | | Asia | | Oceania | | Total | |
| --- | --- | --- | --- | --- | --- | --- | --- | --- | --- | --- | --- | --- | --- | --- |
| **Outside Athletics** |  |  |  |  |  |  |  |  |  |  |  |  |  |  |
| Male |  |  |  |  |  |  |  |  |  |  |  |  |  |  |
| Verbal Abuses | 13.2% | 26.8% | 13.2% | 33.8% | 21.7% | 29.1% | 20.0% | 37.1% | 18.4% | 28.7% | 2.1% | 37.9% | 21.8% | 26.8% |
| Physical Abuses | 1.8% | 9.6% | 8.4% | 26.9% | 11.5% | 17.5% | 10.6% | 25.1% | 10.4% | 19.0% | 2.1% | 37.9% | 12.3% | 16.4% |
| Sexual Abuses | 1.8% | 9.6% | 18.4% | 40.5% | 12.2% | 18.3% | 13.7% | 29.2% | 11.7% | 20.6% | 0.0% | 0.0% | 13.3% | 17.5% |
| Female |  |  |  |  |  |  |  |  |  |  |  |  |  |  |
| Verbal Abuses | 29.0% | 45.3% | 13.2% | 33.8% | 16.2% | 22.9% | 7.7% | 20.9% | 9.1% | 17.3% | 2.1% | 37.9% | 17.5% | 22.2% |
| Physical Abuses | 13.2% | 26.8% | 0.2% | 11.6% | 11.5% | 17.5% | 4.9% | 16.6% | 10.4% | 19.0% | 2.1% | 37.9% | 12.3% | 16.4% |
| Sexual Abuses | 6.1% | 16.8% | 0.0% | 0.0% | 8.2% | 13.5% | 2.3% | 12.0% | 13.0% | 22.3% | 2.1% | 37.9% | 9.8% | 13.5% |
| **Inside Athletics** |  |  |  |  |  |  |  |  |  |  |  |  |  |  |
| Male |  |  |  |  |  |  |  |  |  |  |  |  |  |  |
| Verbal Abuses | 7.7% | 20.9% | 17.6% | 49.0% | 29.7% | 40.2% | 22.7% | 41.3% | 19.2% | 30.8% | 0.0% | 0.0% | 25.4% | 31.7% |
| Physical Abuses | 3.5% | 13.2% | 0.0% | 0.0% | 12.8% | 21.0% | 12.0% | 28.0% | 12.7% | 23.0% | 0.0% | 0.0% | 12.7% | 17.8% |
| Sexual Abuses | 1.0% | 8.1% | 17.6% | 49.0% | 12.8% | 21.0% | 8.7% | 23.3% | 9.6% | 19.0% | 0.0% | 0.0% | 12.3% | 17.3% |
| Female |  |  |  |  |  |  |  |  |  |  |  |  |  |  |
| Verbal Abuses | 47.8% | 66.5% | 17.6% | 49.0% | 17.2% | 26.2% | 8.7% | 23.3% | 11.2% | 21.0% | 14.6% | 85.4% | 20.2% | 26.1% |
| Physical Abuses | 37.8% | 51.1% | 0.0% | 0.0% | 4.4% | 10.1% | 5.5% | 18.5% | 8.1% | 16.9% | 0.0% | 0.0% | 7.8% | 11.9% |
| Sexual Abuses | 92.8% | 107.2% | 0.0% | 0.0% | 0.7% | 4.1% | 0.1% | 7.9% | 9.6% | 19.0% | 14.6% | 85.4% | 6.4% | 10.3% |

Results are expressed as 95% confidence intervals.
