# Supplementary material for: Lifetime Prevalence of Verbal, Physical, and Sexual Abuses in Young Elite Athletics Athletes
Source: Front Sports Act Living. 2021 May 31;3:657624. doi: 10.3389/fspor.2021.657624 (PMC8200562; doi:10.3389/fspor.2021.657624)
Supplement: Supplementary file 2 [file Table_2.DOCX]

**Table 2.** Confidence intervals for prevalence of lifetime sexual abuse experiences outside Athletics displayed by global geographical region.

|  | North America | South America | Europe | Africa | Asia | Oceania | Total |
| --- | --- | --- | --- | --- | --- | --- | --- |
| **Females** |  |  |  |  |  |  |  |
| No-touching sexual abuse | 1.8% - 5.8% | 0% - 0% | 4.3% - 6.8% | 0.6% - 11.2% | 16.4% - 22.8% | 0.6% - 11.2% | 7.3% - 9.2% |
| Touching sexual abuse | 1.8% - 5.8% | 0% - 0% | 5.9% - 8.7% | 0.6% - 11.2% | 3% - 6.9% | 0% - 0% | 4.9% - 6.6% |
| Any sexual abuse | 6.1% - 11.5% | 0% - 0% | 11% - 14.5% | 8.4% - 22.4% | 20.6% - 27.3% | 0.6% - 11.2% | 12.5% - 14.9% |
| **Males** |  |  |  |  |  |  |  |
| No-touching sexual abuse | 0% - 0% | 4.2% - 12.6% | 6.9% - 9.5% | 7.7% - 14.4% | 15.3% - 22.3% | 0% - 0% | 8.4% - 10.4% |
| Touching sexual abuse | 1.8% - 5.8% | 14.2% - 25.1% | 6.9% - 9.5% | 4.9% - 10.8% | 3.7% - 8.4% | 0% - 0% | 7.7% - 9.6% |
| Any sexual abuse | 1.8% - 5.8% | 19.7% - 31.4% | 13.3% - 16.7% | 13.7% - 21.5% | 22.9% - 30.6% | 0% - 0% | 15.5% - 18% |
| **All** |  |  |  |  |  |  |  |
| No-touching sexual abuse | 0.9% - 2.9% | 1.4% - 4.6% | 6.4% - 8.3% | 7.9% - 13.6% | 17.8% - 22.5% | 0.1% - 5.3% | 8.4% - 9.8% |
| Touching sexual abuse | 2.9% - 5.7% | 4.8% - 9.1% | 7.2% - 9.1% | 5.7% - 10.9% | 4.6% - 7.5% | 0% - 0% | 6.9% - 8.1% |
| Any sexual abuse | 5.2% - 8.6% | 6.6% - 11.4% | 13.3% - 15.7% | 14.9% - 21.7% | 23.7% - 28.8% | 0.1% - 5.3% | 14.8% - 16.5% |

Results are expressed as 95% confidence intervals.
