# Supplementary material for: Lifetime Prevalence of Verbal, Physical, and Sexual Abuses in Young Elite Athletics Athletes
Source: Front Sports Act Living. 2021 May 31;3:657624. doi: 10.3389/fspor.2021.657624 (PMC8200562; doi:10.3389/fspor.2021.657624)
Supplement: Supplementary file 3 [file Table_3.DOCX]

**Table 3.** Confidence intervals of lifetime sexual abuse experiences inside Athletics displayed by global geographical region.

| Sexual abuse | North America | South America | Europe | Africa | Asia | Oceania | Total |
| --- | --- | --- | --- | --- | --- | --- | --- |
| **Females** |  |  |  |  |  |  |  |
| No-touching sexual abuse | 1.8% - 5.8% | 0% - 0% | 0% - 1% | 0.6% - 11.2% | 12.4% - 18.2% | 0% - 0% | 3.8% - 5.3% |
| Touching sexual abuse | 6.1% - 11.5% | 0% - 0% | 0% - 1% | 0% - 0% | 0% - 0% | 0.6% - 11.2% | 1.6% - 2.6% |
| Any sexual abuse | 8.4% - 14.3% | 0% - 0% | 0.6% - 1.9% | 0.6% - 11.2% | 12.4% - 18.2% | 0.6% - 11.2% | 5.7% - 7.4% |
| **Males** |  |  |  |  |  |  |  |
| No-touching sexual abuse | 0% - 2.9% | 4.2% - 12.6% | 1.6% - 3.2% | 2.3% - 7.2% | 8.1% - 13.9% | 0% - 0% | 4.1% - 5.6% |
| Touching sexual abuse | 0% - 0% | 4.2% - 12.6% | 5.5% - 8% | 2.3% - 7.2% | 3.7% - 8.4% | 0% - 0% | 5.2% - 6.8% |
| Any sexual abuse | 0% - 2.9% | 9% - 18.8% | 8.3% - 11.1% | 7.7% - 14.4% | 15.3% - 22.3% | 0% - 0% | 9.9% - 12% |
| **All** |  |  |  |  |  |  |  |
| No-touching sexual abuse | 1.9% - 4.3% | 1.4% - 4.6% | 1.2% - 2.2% | 3.6% - 8.2% | 12.1% - 16.3% | 0% - 0% | 4.4% - 5.4% |
| Touching sexual abuse | 2.9% - 5.7% | 1.4% - 4.6% | 3.4% - 4.8% | 1.7% - 5.4% | 1.6% - 3.8% | 0.1% - 5.3% | 3.8% - 4.8% |
| Any sexual abuse | 5.2% - 8.6% | 3% - 6.9% | 5.3% - 7% | 7.9% - 13.6% | 15.5% - 20% | 0.1% - 5.3% | 8.4% - 9.8% |

Results are expressed as 95% confidence intervals.
