# Supplementary material for: Lifetime Prevalence of Verbal, Physical, and Sexual Abuses in Young Elite Athletics Athletes
Source: Front Sports Act Living. 2021 May 31;3:657624. doi: 10.3389/fspor.2021.657624 (PMC8200562; doi:10.3389/fspor.2021.657624)
Supplement: Supplementary file 4 [file Table_4.DOCX]

**Table 4.** Type of sexual abuse outside Athletics (in numbers) displayed by global geographical area.

|  | North America | South America | Europe | Africa | Asia | Oceania | Total |
| --- | --- | --- | --- | --- | --- | --- | --- |
| **Female** |  |  |  |  |  |  |  |
| Exposure | 2 | 0 | 7 | 1 | 10 | 1 | 21 |
| Touched or tried to undress or have sex | 2 | 0 | 7 | 1 | 2 | 0 | 12 |
| Masturbation | 1 | 0 | 1 | 1 | 0 | 0 | 3 |
| Vaginal | 1 | 0 | 0 | 0 | 1 | 0 | 2 |
| Oral | 0 | 0 | 1 | 0 | 0 | 0 | 1 |
| Anal | 0 | 0 | 1 | 0 | 0 | 0 | 1 |
|  | North America | South America | Europe | Africa | Asia | Oceania | Total |
| **Male** |  |  |  |  |  |  |  |
| Exposure | 0 | 2 | 12 | 4 | 8 | 0 | 26 |
| Touched or tried to undress or have sex | 1 | 3 | 3 | 0 | 1 | 0 | 8 |
| Masturbation | 1 | 0 | 4 | 2 | 1 | 0 | 8 |
| Vaginal | 0 | 2 | 4 | 0 | 2 | 0 | 8 |
| Oral | 1 | 3 | 4 | 2 | 0 | 0 | 10 |
| Anal | 0 | 0 | 2 | 1 | 0 | 0 | 3 |
|  | North America | South America | Europe | Africa | Asia | Oceania | Total |
| Female, Male |  |  |  |  |  |  |  |
| Exposure | 2 | 2 | 19 | 5 | 18 | 1 | 47 |
| Touched or tried to undress or have sex | 3 | 3 | 10 | 1 | 3 | 0 | 20 |
| Masturbation | 2 | 0 | 5 | 3 | 1 | 0 | 11 |
| Vaginal | 1 | 2 | 4 | 0 | 3 | 0 | 10 |
| Oral | 1 | 3 | 5 | 2 | 0 | 0 | 11 |
| Anal | 0 | 0 | 3 | 1 | 0 | 0 | 4 |
