# Supplementary material for: Lifetime Prevalence of Verbal, Physical, and Sexual Abuses in Young Elite Athletics Athletes
Source: Front Sports Act Living. 2021 May 31;3:657624. doi: 10.3389/fspor.2021.657624 (PMC8200562; doi:10.3389/fspor.2021.657624)
Supplement: Supplementary file 5 [file Table_5.DOCX]

**Table 5.** Type of sexual abuse inside Athletics (in numbers) displayed by global geographical area.

|  | North America | South America | Europe | Africa | Asia | Oceania | Total |
| --- | --- | --- | --- | --- | --- | --- | --- |
| **Female** |  |  |  |  |  |  |  |
| Exposure | 2 | 0 | 1 | 1 | 8 | 0 | 12 |
| Touched or tried to undress or have sex | 2 | 0 | 1 | 0 | 0 | 0 | 3 |
| Masturbation | 0 | 0 | 0 | 2 | 2 | 0 | 4 |
| Vaginal | 2 | 0 | 0 | 0 | 0 | 1 | 3 |
| Oral | 0 | 0 | 0 | 0 | 0 | 0 | 0 |
| Anal | 0 | 0 | 0 | 0 | 0 | 0 | 0 |
|  | North America | South America | Europe | Africa | Asia | Oceania | Total |
| **Male** |  |  |  |  |  |  |  |
| Exposure | 1 | 2 | 4 | 2 | 5 | 0 | 14 |
| Touched or tried to undress or have sex | 0 | 0 | 4 | 0 | 1 | 0 | 5 |
| Masturbation | 1 | 0 | 5 | 1 | 0 | 0 | 7 |
| Vaginal | 0 | 1 | 3 | 0 | 2 | 0 | 6 |
| Oral | 0 | 1 | 3 | 1 | 0 | 0 | 5 |
| Anal | 0 | 0 | 2 | 1 | 0 | 0 | 3 |
|  | North America | South America | Europe | Africa | Asia | Oceania | Total |
| **Female, Male** |  |  |  |  |  |  |  |
| Exposure | 3 | 2 | 5 | 3 | 13 | 0 | 26 |
| Touched or tried to undress or have sex | 2 | 0 | 5 | 0 | 1 | 0 | 8 |
| Masturbation | 1 | 0 | 5 | 3 | 2 | 0 | 11 |
| Vaginal | 2 | 1 | 3 | 0 | 2 | 1 | 9 |
| Oral | 0 | 1 | 3 | 1 | 0 | 0 | 5 |
| Anal | 0 | 0 | 2 | 1 | 0 | 0 | 3 |
