# Supplementary material for: Lifetime Prevalence of Verbal, Physical, and Sexual Abuses in Young Elite Athletics Athletes
Source: Front Sports Act Living. 2021 May 31;3:657624. doi: 10.3389/fspor.2021.657624 (PMC8200562; doi:10.3389/fspor.2021.657624)
Supplement: Supplementary file 6 [file Table_6.DOCX]

**Questionnaire onwellbeing, health, and experiences of harassment and abuse/ በደህንነት ጤና እንዲሁም በትንኮሳ እና ያለ አግባቡ በሚደርሱ ተፅዕኖዎች ላይ ልምድ/ተሞክሮ ላይ የሚዳስስ መጠይቅ/**

The survey consists of four sections andtakes approximately 5-6 minutes to complete: /

(ይህ የዳሰሳ ጥናት አራት ክፍሎችን ያየዘ ሲሆን በአማካኝ ከ 5-6 ደቂቃ ሞልቶ ለማጠናቀቅ ይፈጃል፡፡)

A - Personal information (1 min) / ሀ- የግልመረጃ (1 ደቂቃ)

B -Your wellbeing (1 min) /ለ- ስለ እራስ ደህንነት (1 ደቂቃ)

C -Your health (1 min) /ሐ- ስለ እራስ ጤና (1 ደቂቃ)

D1, D2 - Experiences of harassment and abuse (3 mins)/ መ1 መ2- ስለ ትንኮሳና ያለ አግባቡ በሚደርሱ ተፅዕኖች ላይ ያለ ልምድ ተመክሮ (3 ደቂቃ)

Please consider following key definitions when you answer the survey: /ከዚህ በታች ያሉትን ቁልፍ ትርጉሞች የዳሰሳ ጥናቱን ሲመልሱ እባክዎን ከግንዛቤ ውስጥ ያስገቡ

**Harassment/ትንኮሳ**

Harassment relates to unwanted attention or conduct, the violation of dignity and/or the creation of a threatening, hostile, intimidating, degrading, humiliating or offensive environment./ ትንኮሳ ከማይፈልግ ሀሳብ ወይም ተግባር ክብርን የሚነካ የተግባር ጥሰት እና/ወይም ማስፈራራት ጠላትነት ማሽማቀቅ ማዋረድ ማሳፈር ወይም አስከፊ አከባቢ /

**Abuse /ያለአግባብ የሚደርስ ተፅእኖ**

Abuse implies that a person’s rights are violated by another. This is based on the abuse of power and trust.

/ያለአግባብ የሚደርስ ተፅእኖ ማለት የሰዎች መብት በሌላ ሲጣስ ይህም ያለ አግባብ ሥልጣን፣ ሀይል እና እምነት በማሳደር መጠቀም ላይ የተመሠተ ነው፡፡/

1. **Personal information / ሀ.**የግልመረጃ
2. How old are you?/እድሜህ/ሽ ስንት ነው Age/እድሜ
3. Sex /ፆታ  Female ሴት

Male ወንድ

1. Which geographical area are you from? /ከየትኛው የመሬት አቀማመጥ ነህ/ሽ

North America/ሰሜን አሜርካ

Central America and Caribbean islands

/መካከለኛው አሜሪካ እና የካረቢየን ደሴት/

South America/ ደቡብ አሜሪካ

Europe/አውሮፓ

Eastern Europe and Caucasus

/ምስራቅ አውሮፓ እና ኳኬሽያን

Northern Africa/ሰሜን አፍሪካ

Central Africa/መካከለኛው አፍሪካ

Southern Africa/ደቡብ አፍሪካ

Middle East /መካከለኛው ምስራቅ

Central Asia/መካከለኛ ኤቨያ

Southern Asia/ደቡብ ኤቪያ

Eastern Asia /ምስራቅ ኤቭያ

South-East Asia/ደቡብ ምራቅ ኤቪያ

Oceania አሽያና

1. How old were you when you started athletics?/ አትሌቲክስ /ሩጫን/ ስትጀምር /ሪ ስንት አመትህ/ሽ ነበር

< 8 years<8 ዓመት  8-12 years/8-12 ዓመት  > 12 years />12 ዓመት

1. To what group events does your main event belong? ከየትኞቹ የአትሌቲክስ ዘረፋ ነው ያንተ/ቺ ዋና ዘረፍ የሚመደበው?  Jumps ዝላይ

Throws ውርወራ

Sprints አጭር ርቀት

Middle/long-distance running

መካከለኛ/ረጅም - ርቀት ሩጫ

Combined events የተዋሀድ ዘርፋዎች

Race walk እርምጃ

1. How many hours on average do you spend training and/or competing in athletics per week?

በአማካኝ ስንት ሰዓት ለልምምድ እና /ወይም በአትሌትክስ በውድድር ላይ ትሆናለህ/ሽ?

Hours/ በሰዓት

**About your wellbeing / ሀ.ስለደህንነትህ/ሽ**

1. Please indicate for each of the five statements, which is closest to how you have been feeling **over the last two weeks.** Notice that higher numbers mean better well-being. Example: If you have felt cheerful and in good spirits more than half of the time during the last two weeks, put a tick in the box with the number 3 in the upper right corner.

/ከዚህ በታች ላሉት ለእያንዳንዱ በዚህ 2 ሳምነት ውስጥ ከሚሰማህ/ሽ ስሜት የሚቀርበውን እባክህን/ሽ ይለፅ/ግለጪ ትላልቅ ቁጥሮች ማለት የተሻለ ደህንነት የሚያመለክት መሆኑን ይገንዘቡ፡፡**ለምሳሌ** ባለፉት ሳምንታት ከግማሽ በላይ ሰዓት ደስተኛ እና በጥሩ መንፈስ ላይ ከነበርክ ነበርሽ በሌላኛው ቀኝ ጥግ ላይ ያለውን ቁጥር 3 የሚለውን ሳጥን ውስጥ ምልዕክት ያድርጉ

|  | Over the last two weeks  ባለፉት 2 ሳምንታትውስጥ | All of the time  በሁሉምጊዜ | Most of the time  አብዛኛውን ጊዜ | More than half of the time  ከግማሽ በላይ ጊዜ | Less than half of the time  ከግማሽ ጊዜ በታች | Some of the time  አንዳንዴ | At no time  በምንም ጊዜ |
| --- | --- | --- | --- | --- | --- | --- | --- |
| **1** | **I have felt cheerful and in good spirits**  ደስተኛ እና ጥሩ ስሜት ተስምቶኝ ነበር | 5 | 4 | 3 | 2 | 1 | 0 |
| **2** | **I have felt calm and relaxed**  የመረጋጋት እና ዘና የማለት ስሜት ተሰምቶኝ ነበር | 5 | 4 | 3 | 2 | 1 | 0 |
| **3** | **I have felt active and vigorous**  መነቃቃት እና ብርቱ ስሜት ተሰምቶኝ ነበር | 5 | 4 | 3 | 2 | 1 | 0 |
| **4** | **I woke up feeling fresh and rested**  የመታደስ እና ያረፈ ስሜት ሰነቃ ተሰምቶኛል | 5 | 4 | 3 | 2 | 1 | 0 |
| **5** | **My daily life has been filled with things that interest me**  በየዕለት ኑሮ በሂወቴ ነገሮች በሚስቡኝ ነገሮች ተሞልተዋል፡፡ | 5 | 4 | 3 | 2 | 1 | 0 |

1. **About your health /** ስለ ጤናዎ/
2. Have you during **the past 12 months**suffered any **sports-related injury** that restricted your normal training? / ባለፉት 12 ወራት ውስጥ የዕለት የልምምድ ስርዓትን የገደበ ከስፖርት ጋር የተያያዘ ጉዳት አጋጥሞህ/ሽ ያውቃል?

Yes አለ

No (🡪question n. 12) የለም (መልሶ የለም ከሆነ ወደ ጥያቄ ቁ.12 ይሂድ)

1. How did the injury first occur? ጉዳቱ መጀመሪያ እንዴት ተከሰተ?

Following a traumatic event, e.g. collision/fall አሰቃቂ አጋጣሚን ተከትሎ ምሳሌ ግጭት/መውደቅ

Sudden onset while training or competing በልምድ ወይም በውድድር ወቅት ላይ በነበረ አጋጣሚ

Gradual onset over several consecutive trainings or competition with no single causative event በተከታታይ በነበሩ ልምምዶች ወይም ውድድሮች በውል መንሴው ያልታወቀ በውድድር በሂደት የመጣ

1. How long did the injury restrict your normal training? ጉዳቱ ከተለመደው ልምምዶ ምን ያህል አገደህ/አገደሽ?

1-7 days 1-7 ቀን  8-21 days 8-21 ቀን  Longer than 21 days /ከ21 ቀን በላይ/

1. Did you consult a sports physician or physiotherapist for the complaint? ለደረሰው ጉዳት የስፖርት ሀኪም ወይም ፒዝዮቴራፒስት አማክረህ /ሽ ነበር?

Yes አዎ

No If not, why? የለም የለም ከሆነ ምክንያቱን ይግለፁ

I preferred managing the issue on my own የተከሰተውን ችግር በእራሴ መፍታት ስለመረጠኩ

My coach could handle the problem ችግሩን አሰልጣኜ መፍታት ስለማችል

I didn’t have any medical support at that time የህክምና ድጋፍ በወቅቱ ስላልነበረኝ

Other ሌላ

1. Have you during **the past 12 months** suffered any **other injury** (unrelated to sports)?

ባለፉት 12 ወራት ውስጥ ሌላ አይነት ጉዳት ደረሶብህ/ሽ ከስፖርት ጋር ያልተያያዘ ያውቃል?

Yes አዎ

No (🡪 question n. 16) የለም መልሶ የለም ከሆነ ወደ ጥያቄ 16 ይሂድ

1. What caused the injury? የጉዳቱ መንስኤ ምንድን ነበር?

An accident, e.g. in traffic አደጋ ምሳሌ - የትራፊክ

Inter-personal violence በራስ የደረሰ የአደጋ

Other ሌላ

1. How long did the injury restrict your normal training? ለምን ያህል ጊዜ ነበር ጉዳቱ ከተለመደው ልምምድህ ያስቆመህ?

1-7 days 1-7 ቀን  8-21 days 8-21 ቀን  Longer than 21 days 21 ቀን በላይ

1. Did you consult a physician or other medical professional for the injury?

ሀኪም ወይም የህክምና ባለሙያ ስለጉዳቱ አማክረው ነበር?

Yes አዎ

No If not, why? የለም የለም ከሆነ ለምን?

I preferred managing the issue on my own የተከሰተውን ችግር በእራሴ መፍታት ስለመረጠኩ

My coach could handle the problem ችግሩን አሰልጣኜ መፍታት ስለማችል

I didn’t have any medical support at that time የህክምና ድጋፍ በወቅቱ ስላልነበረኝ

Other ሌላ

1. **1. Your experiences of harassment and physical abuse**

መ1. በትንኮሳ እና የአካላዊ ጥቃት ያለህን/ያለሽን ልምድ/ተሞክሮ ዙሩያ

1. Has it happened that an adult did any of the following to you, and if so, in what **context and how often**?

አዋቂ የሆነ ሰው ከዚህ በታች የተገለፁትን ድርጊት ተፈፅሞብህ/ሽ የቃል ከሆነ በምን አግባብ እና በምን ያህል ጊዜ

***Inside Athletics*** */በአትሌቲክስ ውስጥ/* ***Outside Athletics/ ከአትሌቲክስ ውጪ***

Never /መቼም/ Sometimes /አንዳንዴ/ Often /አልፎ /

Insulted you/ስድቦህ/አድቦሽ

Obliged you to train against your will/ያለፍላጎትህ/ሽ ልምምድ እንድታደርግ/ጊ ማስገደድ

Threatened to hit you /ለመምታት መዛት

Isolated you from friends/ከጓደኞች/ሽ መለየት

Pushed, shoved or shook you/ ማግፋት ማናወጥ

Threw something at you/የሆነ ነገር መወርወር

Caused you physical pain or harm/አካላዊ ህመም ወይም ጉዳትመድረስ

Hurt you with his/her hands/በእጅ/እጅዋ መጉዳት

Kicked, bit or hit you with his/her fists/ በቡጢ መምታት ማረገጥ መንከስ

Physically attacked you otherwise/አካላዊ ጥቃት አድርሶ

Threatened to harm or harmed

Someone dear to you/አንተን/አንቺን ለመጎዳት ወይም የምትወደውን ሰው ለመጎዳት ማስፈራራት/ዛቻ

1. If all answers are negative🡪 question n. 20. ሁሉም መልሶዎት የለም ከሆነ ወደ ጥያቄ ቁ.20 ይሂድ

How old were you the first time it happened?/የመጀመርያ ጊዜ ሲከሰት ስንት ዓመትህ ነበር?

Years ዓመት

18) Who did that to you? ምን ነው ይህን ያደረግብህ?

*Several answers may be marked* ከአንድ በላይ መልሶችን መልክት ማድረግ ይቻላል

*.* Parent (Biological father/mother, stepfather/stepmother)/ ወላጅ በተፈጥሮ /በደም የሚዛመድ አባት/እናት እንጀራ አባት/እናት

Siblings (biological/step-siblings) ወንድሞችና እህቶች (የተፈጠሮ/በደምየሚዛመድ)

Other relative ሌላ ዘመድ

Friend or acquaintance to you ጓደኛ ወይም የምታውቀው ሰው

Your partner (boyfriend/girlfriend) ፍቅረኛ (የወንድጓደኛ/የሴትጓደኛ)

Other athlete ሌላ አትሌት

Athletic trainer, Coach, Medical staff የአትሌቲክ አላማማጅ አሰልጣኝ የህክምና ሠራተኛ

Teacher አስተማሪ

Someone totally unknown ሌላ የማይታወቅ ሰው

1. Did you see a physician or counselor in response to what has happened to you? በደረሰቦት ጉዳይ ላይ ሀኪም ወይም አማካሪ አይተው ያቃሉ

Yesአዎ

No, there was no reason የለም ምንም አይነት ምክንያት አለበረኝም

No, but I now believe that I should have የለም ግን አሁን ማድረግ ነበረብኝ ብዬ አምናለሁ

**D.2. your experiences of sexual abuse /** መ. 2 ከወስባዊ ጥቃት ጋር በተያያዘ ያጋጠሙ ሁኔታዎች

1. Have you **ever** been persuaded, pushed or forced into sexual acts against your will in your life, **outside of athletics**?

በሂወትዎ ያለ ፈቃድዎ ከአትሌቲክስ ውጪ በወሲባዊ ድርጊት እንዲፈፀሙ በማሳመን የመገፋት ወይም የመገደድ ያለፍላጎቶ ደርሶበት ያውቃል?

*Several answers may be marked* ከአንድ በላይ መልሶችን መልክት ማድረግ ይቻላል

I have not been subjected to the above against my will (🡪 End survey) ያለፈላጎቴ ከላይ የተጠቀሰውን ተግባር ሆኖብኝ አያውቅም (---የዳሰሳ ጥናት ማለቂያ)

Someone has exposed himself/herself to you ሌላ ወንድ/ሴት እራሱን አጋልጦ አሳውቆኝ ያውቃል

Someone has touched your genitals or tried to undress you, to have sex with you

ሌላ ሰው ግብረ ስጋ ግንኙነት ለማድረግ የማራብያ አካሎትን የመነካካት ወይም ልብሶትን ለማውለቅ ሙከራ አድርገዋል

You have masturbated for someone

ወሲብ እንዲፈፀሙ ለሌላ ሰው ብልትን በመነሳሳት ዘርን አፍስሶ ስሜቱ እንዲረካ አድርገዋል

You have had vaginal intercourse ከሴት ብልት ጋር ግንኙነት አድርገዋል

You have had oral sex በአፍ በኩል የሚደረግ ወሲብ ፈፅመው ያውቃሉ

You have had anal sex በፊንጢጣ በኩል የሚደረግ ወሲብ ፈፅመዋል

1. How many times did that happen? /ምን ያህል ጊዜ ነበር የተከሰተው

Once አንዴ

2-5 times ከ2-5 ጊዜ

More than 5 times ከ5 ጊዜ በላይ

1. How old were you the first time you experienced sexual abuse? ለመጀመርያ ጊዜ የወሲባዊ ጥቃት ሲደርስብህ እድሜዎ ስንት ነበር?

Years/ እድሜ

1. Have you ever been persuaded, pushed or forced into sexual acts against your will, **in connection with athletic activities or gatherings**?

ከአትሌቲክስ ጋር በተያያዘ በሂወትዎ ያለ ፈቃድዎ በወሲባዊ ድርጊት እንዲፈፀሙ በማሳመን የመገፋት ወይም የመገደድ ያለፍላጎቶ ደርሶበት ያውቃል?

*Several answers may be marked* ከአንድ በላይ መልሶችን መልክት ማድረግ ይቻላል

I have not been subjected to the above against my will (🡪 End survey) ያለፈላጎቴ ከላይ የተጠቀሰውን ተግባር ሆኖብኝ አያውቅም (---የዳሰሳ ጥናት ማለቂያ)

Someone has exposed himself/herself to you ሌላ ወንድ/ሴት እራሱን አጋልጦ አሳውቆኝ ያውቃል

Someone has touched your genitals or tried to undress you, to have sex with you ሌላ ሰው ግብረ ስጋ ግንኙነት ለማድረግ የማራብያ አካሎትን የመነካካት ወይም ልብሶትን ለማውለቅ ሙከራ አድርገዋል

You have masturbated for someone ወሲብ እንዲፈፀሙ ለሌላ ሰው ብልትን በመነሳሳት ዘርን አፍስሶ ስሜቱ እንዲረካ አድርገዋል

You have had vaginal intercourse ከሴት ብልት ጋር ግንኙነት አድርገዋል

You have had oral sex በአፍ በኩል የሚደረግ ወሲብ ፈፅመው ያውቃሉ

You have had anal sex በፊንጢጣ በኩል የሚደረግ ወሲብ ፈፅመዋል

1. How old were you the first time you experienced sexual abuse? ለመጀመርያ ጊዜ የወሲባዊ ጥቃት ሲደርስብህ እድሜዎ ስንት ነበር?

Years/ እድሜ

1. Who did that to you? ምን ነው ይህን ያደረግብህ?

*Several answers may be marked* ከአንድ በላይ መልሶችን መልክት ማድረግ ይቻላል

*.* Parent (Biological father/mother, stepfather/stepmother)/ ወላጅ በተፈጥሮ /በደም የሚዛመድ አባት/እናት እንጀራ አባት/እናት

Siblings (biological/step-siblings) ወንድሞችና እህቶች (የተፈጠሮ/በደምየሚዛመድ)

Other relative ሌላ ዘመድ

Friend or acquaintance to you ጓደኛ ወይም የምታውቀው ሰው

Your partner (boyfriend/girlfriend) ፍቅረኛ (የወንድጓደኛ/የሴትጓደኛ)

Other athlete ሌላ አትሌት

Athletic trainer, Coach, Medical staff የአትሌቲክ አላማማጅ አሰልጣኝ የህክምና ሠራተኛ

Teacher አስተማሪ

Someone totally unknown ሌላ የማይታወቅ ሰው

1. Did you see a physician or counselor in response to what has happened to you? በደረሰቦት ጉዳይ ላይ ሀኪም ወይም አማካሪ አይተው ያቃሉ

Yesአዎ

No, there was no reason የለም ምንም አይነት ምክንያት አለበረኝም

No, but I now believe that I should have የለም ግን አሁን ማድረግ ነበረብኝ

1. Were you drunk or drugged the first time it happened in the context of athletics activities or gatherings? ከአትሌቲክስ ድርጊት ወይም መስባሰብ ጋር በተያያዘ ለመጀመሪያ ጊዜ ድርጊቱ ሲፈፀም ጠጥተው ወይም መድሀኒት ተደርጓበት ነበር?

Yes/ አዎ

No/የለም

1. What forms of persuasion, pressure or force, did the person in question, usein connection with athletic activities or gatherings? *Several answers may be marked*.

አትሌቲክስ ድርጊት ወይም መሰባሰብ ጥያቄ ጋር በተያያዘ ድርጊቱን የፈፀመው ሰው በምን አይነት የማሳመኛ መንገድ/ጫና ወይም የሀይል መንገድ ነው?ከአንድ በላይ መልስ ላይ ምልዕክት ማድረግ ይቻላል

Fooled youማታለል

Abused his positionያለውን የስልጣን ቦታ በመጠቀም

Persuaded you አሳምኖት

Threatened to reject you ላለመቀበል በማስፈራራት

Held you በመያዝ

Hit you or hurt you በመምታት ወይመ መጉዳት

Provided alcohol, drugs or tabletsአልኮል መድሀኒት ወይም እንክብሎችንበመስጠት

Otherሌላ

1. Did the person in question try to compensate you in the form of gifts, money, etc.?

ተጠያቂው ሰው በስጦታ ወይም በገንዘብ መልክ ለመካስ ሞክሮ ነበር

Yes አዎ

No የለም

1. Have you ever sought help or support in respect to:

ለሚከተሉት እርዳታ ወይም ድጋፍ ፈልገው ያውቃሉ

Yes አዎ No የለም

- Being a victim of psychological abuseየስነ ልቦና ጥቃት ሰለባ ሆነው
- Being a victim of physical abuseየአካላዊ ጥቃት ሰለባ ሆነው

- Being a victim of sexual abuse የወሲባዊ ጥቃት ሰለባ ሆነው

- Reporting someone for committing sexual abuse ወሲባዊ ጥቃት የፈፀመን ሰው አጋልጠው

- Having problems with parents ከወላጆች ጋር ችግር መኖር

- Experiencing mental health problems የአእምሮ ጤና ችግር ማጋጠም

- Other ሌላ

1. Whomdid you ask for help?

ከማንነው እርዳታ የጠየቁት?

*Several answers may be marked* ከአንድ በላይ መልስ ላይ ምልዕክት ማድረግ ይቻላል

Parents ወላጆች

Siblings እህት/ወንድም

Girlfriend / Boyfriend የሴትጓደኛ/የወንድጓደኛ

Same aged friend ተመሳሳይ እድሜ ላይ ያለ ጓደኛ

Adult relative or friend አዋቂ ዘመድ ወይም ጓደኛ

"Professional" - teachers, counselors, social support, nurse or equivalent “ባለሙያ” -አስተማረ አማካሪ የማህበረሰብ ድጋፈ ሰጪ ነርስ ወይም ተመሳሳይ

"Athletics official" - coaches, club official, or equivalent የአትሌቲክስ አመራር አሰልጣኝ የክለብ አመራር ወይም ተመሳሳይ

Other person ሌላ ሰው

It was reported to social services or the police ለማህበረሰብ አገልግሎት ወይም ለፖሊስ ሪፖርት ተደርጉል

1. Did you receive the support and the help that you needed? በወቅቱ የሚያሰፈልጉትን ድጋፍ እና እርዳታ አግኝተው ነበር

Yes አዎ

No የለም

1. If you have reported episodes of harassment and/or abuse, are you satisfied with the way this was dealt with?

የተለያዩ የተፈፀሙ ትንኮሳዎች እና /ወይም ጥቃት አመልክተው ከሆነ ጉዳዩ የተኬሄደበት መንገድ እርሶዎን አጥግቦታል፡፡

Yes አዎ

No የለም

1. Are you aware of any safeguarding policy or code of conduct implemented by your National Federation? በብሔራዊ ፌድሬሽን ድርጊቱ እንዳይፎፀም ማንኛውም አይነት የማስጠበቂያ ፖሊሲ ወይም የህግ ደምብ እንደሚተገበር ማስፈፀሚያ መኖሩን ያውቃሉ

Yes አዎ

No የለም

Send your data by pressing the Submit button./ የሞሉትን መረጃ ያስተላልፉ የሚለውን ላይ በመጫን ያስተላልፉ/
