# Supplementary material for: Lifetime Prevalence of Verbal, Physical, and Sexual Abuses in Young Elite Athletics Athletes
Source: Front Sports Act Living. 2021 May 31;3:657624. doi: 10.3389/fspor.2021.657624 (PMC8200562; doi:10.3389/fspor.2021.657624)
Supplement: Supplementary file 7 [file Table_7.DOCX]

**幸福感、健康、ハラスメントや虐待の経験に関する質問表**

このアンケート調査は4項目より構成され、５～６分で終了できます。

A　個人情報（1分）

B　あなたの幸福感（1分）

C　あなたの健康（1分）

D1, D2　ハラスメントや虐待の経験（3分）

このアンケート調査は回答される際に、次の用語の定義をご参照ください。

**ハラスメント**

ハラスメントとは、望まなかった対応や行為、尊厳の侵害、および／もしくは脅迫的、敵対的、おびえさせる、品位を下げる、屈辱を与える、もしくは攻撃的な状況を作ること、を指します。

**虐待**

虐待とは、個人の権利が他者によって妨害されることを指します。権力や信頼の悪用に基づきます。

**個人情報**

1. 年齢をお答えください。　　年齢　 Age

1. 性別  女性

男性

1. 出身地域をお答えください。

北米

中央アメリカおよびカリブ地方

南アメリカ

ヨーロッパ

東ヨーロッパおよびコーカサス

北アフリカ

中央アフリカ

南アフリカ

中近東

中央アジア

南アジア

東アジア

東南アジア

オセアニア

1. 陸上競技を始めた年齢はおいくつでしたか。  < 8 歳未満 　 8-12 歳 　 > 13 歳以上
2. どの競技種目にあなたの種目は入りますか

跳躍

投擲

短距離

中長距離

混成

競歩

1. 1週間の間に、陸上競技の練習や競技会に平均してどれくらいの時間を使っていますか

時間

1. **あなたの幸福感**
2. 次の5つの項目について、あなたが過去2週間において感じていたものに、最も近いものにチェックしてください。数字が大きいほど、幸福感が高いことを意味します。たとえば、過去2週間の半分以上で、機嫌が良く、かつ良い気力を感じていたら、一番上の段に３と数字が書かれたところをチェックしてください。

|  | 過去2週間において | いつも | ほとんど | 半分以上 | 半分以下 | ときどき | 全くない |
| --- | --- | --- | --- | --- | --- | --- | --- |
| **1** | **機嫌が良く、かつ良い気力を感じていた** | 5 | 4 | 3 | 2 | 1 | 0 |
| **2** | **落ち着いていて、リラックスしていた** | 5 | 4 | 3 | 2 | 1 | 0 |
| **3** | **積極的で、元気であった** | 5 | 4 | 3 | 2 | 1 | 0 |
| **4** | **よく休めて、元気を感じながら起床した** | 5 | 4 | 3 | 2 | 1 | 0 |
| **5** | **My daily life has been filled with things that interest me** | 5 | 4 | 3 | 2 | 1 | 0 |

1. **あなたの健康について**
2. **過去12ヶ月**に、通常の練習に制限があった**スポーツ関連傷害（ケガ）**がありましたか？

はい

いいえ（質問12へ進む）

1. そのケガは最初どのように起こりましたか？

衝突や転倒などの外傷の後

練習中もしくは競技中に突然発生した

1度の事ではなく、反復する練習や競技で徐々に起こった

1. どこくらいの期間、ケガによって通常の練習に制限がありましたか？

1-7 日

8-21 日

22日以上

1. 症状についてスポーツドクターやトレーナーに相談しましたか？

はい、しました。

いいえ、

それは、どうしてでしょうか？

自分一人で対応したかった

自分のコーチが対応してくれた

その時はメディカルサポートを受けていなかった

その他

1. **過去12ヶ月**に、**スポーツに関連しない**他の傷害（ケガ）がありましたか？

はい、ありました

いいえ（質問16へ進む）

1. ケガの原因は何でしたか？

交通事故などの事故

他の人による暴力

その他

1. どのくらいの期間、ケガによって通常の練習に制限がありましたか？

1-7 日

8-21 日

22日以上

1. ケガについてドクターや医療関係者に相談しましたか？

はい、しました。

いいえ、

それは、どうしてでしょうか？

自分一人で対応したかった

自分のコーチが対応してくれた

その時はメディカルサポートを受けていなかった

その他

1. **1. ハラスメントや身体的虐待についての経験**
2. 大人の人が、あなたに対して次の行為をしましたか？　それは**どのような状況**で、**頻度**はどうでしたか？

**陸上競技内で**　　　　　　　**陸上競技外で**

**全くない　ときどき　たびたび　　全くない　ときどき　たびたび**

侮辱された

あなたの意思に反して練習　　　　

を強制された

殴るとおどかされた

あなたの友達から孤立させられた

押されたり、突かれたり、

揺さぶられた

何かを投げつけられた　　　　　　

身体的痛みや危害が加えられた

その人の手で傷つけられた　　　　

蹴られたり、嚙まれたり、　　　

げんこつで殴られた

それ以外で、身体に暴行された　　

あなたが好きな人を傷つけると

脅かされる、もしくは傷つけられた

すべて　全くない　であれば、質問20へ進む

1. それが最初に起きたのは、何歳の時でしたか？　　　　 年齢(歳)
2. 誰があなたに対してそのようにしましたか？

*複数回答可能*

両親（実の父親／母親、まま父／まま母）

兄弟姉妹（実の兄弟姉妹、まま兄弟姉妹）

それ以外の親族

友人もしくは知人

パートナー（ボーイフレンド、ガールフレンド）

他のアスリート

トレーナー、コーチ、医療スタッフ

教師

全く知らない人

1. あなたに起きたことに対して、医師もしくはカウンセラーを受診しましたか？

はい、

いいえ、理由はありません。

いいえ、しかし、今はそうすべきだったと思います。

**D.2. 性的虐待についての経験**

1. **陸上競技外で**、あなたの意思に反して、性的行為をさせられたり、おしつけられたり、または強要させられたりしたことが、**これまでに**ありましたか？　下記についてお答えください。

*複数回答可能*

自分の意思に反して、上記の対象になっていません。（これで調査は終了します）

誰かが陰部をあなたに露出した。

誰かがあなたの陰部に触れた、もしくはセックスするためにあなたを裸にしようとした。

あなたは誰かを自慰行為しました。

性行為をした。

オーラルセックスをした。

肛門セックスをした。

1. それは何度ありましたか？  一度

2-5 回

6回以上

1. 性的虐待が最初にあったのは、何歳でしたか？ 年齢（歳）

1. **陸上競技活動に関連して**、あなたの意思に反して、性的行為をさせられたり、おしつけられたり、または強要させられたりしたことが、これまでにありましたか？

*複数回答可能*

自分の意思に反して、上記の対象になっていません。（これで調査は終了します）

誰かが陰部をあなたに露出した。

誰かがあなたの陰部に触れた、もしくはセックスするためにあなたを裸にしようとした。

あなたは誰かを自慰行為しました。

性行為をした。

オーラルセックスをした。

肛門セックスをした。

1. 性的虐待が最初にあったのは、何歳でしたか？ 年齢（歳）
2. 誰があなたに対して、そのようにしましたか？

*複数回答可能*

両親（実の父親／母親、まま父／まま母）

兄弟姉妹（実の兄弟姉妹、まま兄弟姉妹）

Other relative

友人もしくは知人

パートナー（ボーイフレンド、ガールフレンド）

他のアスリート

トレーナー、コーチ、医療スタッフ

教師

全く知らない人

1. あなたに起きたことに対して、医師もしくは専門家を受診しましたか？

はい、しました。

いいえ、理由はありません。

いいえ、しかし、今はそうすべきだったと思います。

1. 陸上競技活動に関連して初めてそのようなことが起きた時、あなたは酔っぱらったりドラッグを使ったりしていましたか？

はい

いいえ

1. 陸上競技活動に関連して、その問題の人物は、どのような形で、性的行為をさせたり、おしつけたり、または強要しましたか？

複数回答可能

あなたをだました

地位を乱用した

あなたを説得させた

あなたを拒絶すると脅した

あなたを拘束した

たたき、傷つけた

アルコール、ドラッグ、錠剤を準備した

その他

1. その問題の人物は、贈り物や金銭などで、あなたに償おうとしましたか？

はい

いいえ

1. 下記について援助や支援を求めましたか？

はい いいえ

心理的虐待の被害者として

身体的虐待の被害者として

性的虐待の被害者として

性的虐待があったことを誰かに報告する　　

両親と問題を共有する

精神的問題を経験した　　　　　　　　　　　　　　

その他

1. 誰に援助をお願いしましたか？

複数回答可能  両親

兄弟姉妹

ガールフレンド／ボーイフレンド

同年齢の友人

成人の親族や友人

“専門家”－教師、カウンセラー、社会的支援、看護師など

“陸上競技関係者”－コーチ、クラブ役員など

その他の人

警察などの公権に報告された

1. あなたが必要としていた援助や支援を受けられましたか？

はい

いいえ

1. あなたがハラスメントや虐待の出来事を報告しているならば、それの取り扱われ方についてあなたは満足していますか？

はい

いいえ

1. あなたは競技団体によって実施されている予防手段や行動規範について知っていますか？

はい

いいえ

提出ボタンを押して、データを送ってください。
