# Supplementary material for: Lifetime Prevalence of Verbal, Physical, and Sexual Abuses in Young Elite Athletics Athletes
Source: Front Sports Act Living. 2021 May 31;3:657624. doi: 10.3389/fspor.2021.657624 (PMC8200562; doi:10.3389/fspor.2021.657624)
Supplement: Supplementary file 8 [file Table_8.DOCX]

**Étude sur le bien-être, la santé et les expériences de harcèlement et de maltraitance**

Ce questionnaire comporte quatre sections. Il peut être rempli en 5 à 6 minutes environ:

A - Renseignements personnels (1 min)

B - Votre bien-être (1 min)

C - Votre santé (1 min)

D 1, D2 – Expériences de harcèlement et de maltraitance (3 min)

Veuillez tenir compte des définitions clés suivantes pour répondre au questionnaire:

**Le harcèlement**

Le harcèlement se définit comme une attention ou une conduite non désirée, une atteinte à la dignité et/ou la création d'un environnement menaçant, hostile, dégradant, humiliant, offensant ou d’intimidation.

**La maltraitance /l’abus**

La maltraitance implique que les droits d’une personne sont violés par une autre personne ; elle est fondée sur l’abus de pouvoir et de confiance.

**Renseignements personnels**

1. Quel âge avez-vous? Age
2. Sexe  Féminin

Masculin

1. De quelle région géographique êtes-vous originaire ?  Amérique du Nord

Amérique centrale et les Caraïbes

Amérique du Sud

Europe

Europe de l’Est et Caucase

Afrique du Nord

Afrique centrale

Afrique australe

Moyen-Orient

Asie centrale

Asie du Sud

Asie de l’Est

Asie du Sud-Est

Océanie

1. Quel âge aviez-vous lorsque vous avez commencé l’athlétisme?  < 8 ans  8-12 ans  > 12 ans
2. A quel groupe d’épreuves votre discipline principale appartient-elle?

Les sauts

 Les lancers

Les sprints

Les courses de demi-fond et de fond

Les épreuves combinées

La marche athlétique

1. Combien d'heures en moyenne consacrez-vous à l'entraînement et/ou à la compétition en athlétisme par semaine ?

Heures

1. **Concernant votre bien-être**
2. Veuillez indiquer pour chacune des cinq affirmations ci-dessous, celle qui se rapproche le plus de votre ressenti **au cours des deux dernières semaines**. Les chiffres les plus élevés signifient un bien-être plus grand. Par exemple: Si vous vous sentiez de bonne humeur et d’humeur joyeuse plus de la moitié du temps au cours des deux dernières semaines, dans ce cas, cochez la case correspondant au chiffre 3.

|  | Au cours des deux dernières semaines | Tout le temps | La plupart du temps | Plus de la moitié du temps | Moins de la moitié du temps | De temps à autre | A aucun moment |
| --- | --- | --- | --- | --- | --- | --- | --- |
| **1** | **J’étais (se) et de bonne humeur** | 5 | 4 | 3 | 2 | 1 | 0 |
| **2** | **J’étais calme et détendu(e)** | 5 | 4 | 3 | 2 | 1 | 0 |
| **3** | **J’étais actif(ve) et dynamique** | 5 | 4 | 3 | 2 | 1 | 0 |
| **4** | **Je me suis réveillé(e) frais(che) et reposé(e)** | 5 | 4 | 3 | 2 | 1 | 0 |
| **5** | **Ma vie quotidienne a été remplie de choses qui m’intéressent** | 5 | 4 | 3 | 2 | 1 | 0 |

1. **Concernant votre santé**
2. **Au cours des 12 derniers mois**, avez-vous subi **des blessures liées à la pratique sportive** ayant limité votre entraînement normal ?

Oui

Non (🡪 question n. 12)

1. Comment la blessure est-elle survenue la première fois ?

Suite à un traumatisme, par ex. collision/chute

Soudainement pendant l’entraînement ou la compétition

Progressivement lors de divers entraînements consécutifs ou des compétitions, sans cause unique l’ayant provoquée.

1. Pendant combien de temps la blessure a-t-elle limité votre entraînement normal ?

1-7 jours

8-21 jours

Plus de 21 jours

1. Avez-vous consulté un médecin ou un physiothérapeute du sport pour cette blessure?

Oui

Non Dans ce cas, pourquoi ?

J’ai préféré gérer le problème moi-même

Mon entraîneur a pu s’occuper du problème

Je n’avais pas d’aide médicale à ce moment-là

Autre raison

1. **Au cours des 12 derniers mois**, avez-vous souffert de toute autre blessure (non liée au sport)?

Oui

Non (🡪 question n. 16)

1. Comment est survenue cette blessure?

Un accident, par ex. de circulation

Violence interpersonnelle

Autre raison

1. Pendant combien de temps la blessure a-t-elle limité votre entraînement normal ?

1-7 jours

8-21 jours

Plus de 21 jours

1. Avez-vous consulté un médecin ou un physiothérapeute du sport pour cette blessure?

Oui

Non Dans ce cas, pourquoi?

J’ai préféré gérer le problème moi-même

Mon entraîneur a pu s’occuper du problème

Je n’avais pas d’aide médicale à ce moment-là

Autre raison

1. **1. Vos expériences de harcèlement et de maltraitance physique**
2. Est-il arrivé qu'un adulte vous ait fait l'une des choses suivantes, et si oui, dans quel **contexte et à quelle fréquence** ?

***Au sein de l’athlétisme*** ***En dehors de l’athlétisme***

Jamais Parfois Souvent Jamais Parfois Souvent

Il vous a insulté(e)

Il vous a obligé(e) à vous entraîner

contre votre gré

Il a menacé de vous frapper

Il vous a isolé(e) de vos amis

Il vous a poussé(e), bousculé(e)

ou secoué(e)

Il vous a jeté quelque chose

Il vous a fait mal/blessé(e) physiquement

Il vous a fait mal avec ses mains

Il vous a frappé(e), mordu(e)

ou donné des coups de poings/pieds

Il vous a attaqué(e) physiquement

d’une autre manière

Il a menacé de faire du mal,

ou il a fait du mal, à quelqu’un qui vous est cher

Si toutes les réponses sont négatives 🡪 question n. 20.

1. Quel âge aviez-vous la première fois que cela s’est produit ? Ans
2. Qui vous a fait cela?

*Vous pouvez cocher plusieurs réponses* Parent (père/mère biologique, beau-père/belle-mère

Fratrie (biologique/par alliance)

Autre membre de la famille

Ami ou connaissance

Votre partenaire (petit ami/petite amie)

Autre athlète

Entraîneur d’athlétisme, Entraîneur, Personnel médical

Enseignant

Quelqu’un d’inconnu

1. Avez-vous consulté un médecin ou un thérapeute suite à ce qui vous est arrivé ?

Oui

Non, il n’y avait aucune raison

Non, mais maintenant je pense que j’aurais dû le faire

**D.2. Vos expériences d’abus sexuel**

1. Avez-vous **déjà** été persuadé(e), poussé(e) ou forcé(e) à des actes sexuels contre votre volonté dans votre vie, **en dehors de l’athlétisme**?

*Vous pouvez cocher plusieurs réponses*

Je n’ai pas fait l’objet de tels actes contre mon gré (🡪 Fin du questionnaire)

Quelqu'un s'est exhibé devant vous

Quelqu'un a touché vos parties génitales ou a essayé de vous déshabiller, d'avoir des rapports sexuels avec vous.

Vous avez masturbé quelqu’un

Vous avez eu des rapports sexuels vaginaux

Vous avez eu des rapports sexuels oraux

Vous avez eu des rapports sexuels anaux

1. Combien de fois cela s'est-il produit?  Une fois

2 à 5 fois

Plus de 5 fois

1. Quel âge aviez-vous la première fois que vous avez été victime d'abus sexuel? Ans
2. Avez-vous **déjà** été persuadé(e), contraint(e) ou forcé(e) à des actes sexuels contre votre gré, **dans le cadre d’activités ou de compétitions en athlétisme ?**

*Vous pouvez cocher plusieurs réponses*

Je n'ai pas subi ce qui précède contre mon gré (🡪 Fin du questionnaire)

Quelqu'un s'est exhibé devant vous.

Quelqu'un a touché vos parties génitales ou a essayé de vous déshabiller, d'avoir des rapports sexuels avec vous.

Vous avez masturbé quelqu’un

Vous avez eu des rapports sexuels vaginaux.

Vous avez eu des rapports sexuels oraux

Vous avez eu des rapports sexuels anaux

1. Quel âge aviez-vous la première fois que vous avez été victime d'abus sexuel? Ans
2. Qui vous a fait cela?

*Vous pouvez cocher plusieurs réponses* Parent (père/mère biologique, beau-père/belle-mère

Fratrie (biologique/par alliance)

Autre membre de la famille

Ami ou connaissance

Votre partenaire (petit ami/petite amie)

Autre athlète

Entraîneur d’athlétisme, Entraîneur, Personnel médical

Enseignant

Quelqu’un d’inconnu

1. Avez-vous consulté un médecin ou une autorité compétente suite à ce qui vous est arrivé?

Oui

Non, il n’y avait pas de raison

Non, mais maintenant je pense que j’aurais dû le faire

1. Etiez-vous ivre ou drogué(e) la première fois que cela s'est produit dans le cadre d'activités ou de compétitions en athlétisme ?  Oui

Non

1. Quelles formes de persuasion, de pression ou de force la personne en question a-t-elle utilisées/exercées dans le cadre d'activités ou compétitions en athlétisme? *Vous pouvez cocher plusieurs réponses*.

Cette personne vous a dupé(e)

Cette personne a abusé de sa position

Cette personne vous a persuadée

Cette personne a menacé de vous rejeter

Cette personne vous a agrippé(e)

Cette personne vous a frappé(e) ou blessé(e)

Cette personne vous a donné de l’alcool, de la drogue, ou des comprimés

Autre

1. La personne en question a-t-elle essayé de vous dédommager sous forme de cadeaux, de sommes d'argent, etc.

Oui

Non

1. Avez-vous déjà demandé de l'aide ou soutien, en relation avec les situations suivantes:

Oui Non

Pour avoir été victime de violence psychologique

Pour avoir été victime de violence physique

Pour avoir été victime de sévices sexuels

Dénoncer quelqu'un ayant commis des abus sexuels

Pour des problèmes avec les parents

Pour des problèmes de santé mentale

Autre

1. A qui avez-vous demandé de l'aide?

*Vous pouvez cocher plusieurs cases*  Parents

Fratrie

Petite amie / Petit ami

Un ami du même âge

Parent ou ami adulte

«Professionnel» - enseignants, conseillers, assistant(e) social(e), infirmière ou équivalent.

"Un Officiel de l’athlétisme" - entraîneur, officiel de club ou équivalent

Autre personne

Cela a été signalé aux services sociaux ou à la police

1. Avez-vous reçu le soutien et l'aide dont vous aviez besoin ?

Oui

Non

1. Si vous avez signalé des actes de harcèlement et/ou de maltraitance, êtes-vous satisfait(e) de la manière dont cela a été traité?

Oui

Non

1. Avez-vous connaissance d'une politique de protection ou d'un code de conduite mis en place par votre Fédération nationale ?

Oui

Non

Envoyer vos réponses en appuyant sur le bouton “Envoyer ” (« Submit »).
