# Supplementary material for: Lifetime Prevalence of Verbal, Physical, and Sexual Abuses in Young Elite Athletics Athletes
Source: Front Sports Act Living. 2021 May 31;3:657624. doi: 10.3389/fspor.2021.657624 (PMC8200562; doi:10.3389/fspor.2021.657624)
Supplement: Supplementary file 9 [file Table_9.DOCX]

**Cuestionario sobre bienestar, salud y experiencias de acoso y abuso**

La encuesta consta de cuatro secciones y se tarda unos 5-6 minutos en completarla:

A - Información personal (1 min.)

B - Su bienestar (1 min.)

C - Su salud (1 min.)

D1, D2 - Experiencias de acoso y abuso (3 minutos)

Tenga en cuenta seguir las definiciones clave cuando responda la encuesta:

**Acoso**

El acoso trata de una atención o conducta no deseada, la violación de la dignidad y/o la creación de un ambiente amenazante, hostil, intimidante, degradante, humillante u ofensivo.

**Abuso**

El abuso implica que una persona viola los derechos de otra. Se basa en el abuso de poder y confianza.

**Información personal**

1. ¿Cuántos años tiene? Años
2. Sexo  Femenino

Masculino

1. ¿Cuál es su área geográfica de procedencia?  Norteamérica

Centroamérica y las islas del Caribe

Sudamérica

Europa

Europa del Este y el Cáucaso

Norte de África

África Central

África del Sur

Oriente Medio

Asia Central

Asia Meridional

Asia Oriental

Sudeste Asiático

Oceanía

1. ¿Qué edad tenía cuando empezó a practicar atletismo?  <8 años  8-12 años  > 12 años
2. ¿A qué tipo de pruebas corresponde su prueba principal?

Salto

Lanzamientos

Carreras

Carreras de media/larga distancia

Pruebas combinadas

Marcha atlética

1. ¿Cuántas horas de media entrena y/o compite en atletismo a la semana?

Horas

1. **En relación con su bienestar**
2. Indique para cada uno de los cinco enunciados cuál es el más parecido a cómo se ha sentido **en las últimas dos semanas**. Tenga en cuenta que las cifras más altas significan un mayor bienestar. Ejemplo: Si ha estado alegre y de buen humor más de la mitad del tiempo durante las últimas dos semanas, marque la casilla con el número 3 en la esquina superior derecha.

|  | En las últimas dos semanas | Todo el tiempo | La mayor parte del tiempo | Más de la mitad del tiempo | Menos de la mitad del tiempo | En algunos momentos | En ningún momento |
| --- | --- | --- | --- | --- | --- | --- | --- |
| **1** | **He estado alegre y de buen humor** | 5 | 4 | 3 | 2 | 1 | 0 |
| **2** | **He estado tranquilo y relajado** | 5 | 4 | 3 | 2 | 1 | 0 |
| **3** | **He estado activo y vigoroso** | 5 | 4 | 3 | 2 | 1 | 0 |
| **4** | **Me desperté sintiéndome fresco y descansado** | 5 | 4 | 3 | 2 | 1 | 0 |
| **5** | **Mi vida diaria está llena de cosas que me interesan** | 5 | 4 | 3 | 2 | 1 | 0 |

1. **En relación con su salud**
2. ¿Ha sufrido alguna **lesión relacionada con el deporte** que haya limitado su entrenamiento normal durante **los últimos 12 meses**?

Sí

No (🡪 pregunta n.° 12)

1. ¿Cómo se produjo la lesión por primera vez?

Después de un evento traumático, por ejemplo, colisión/caída

Inicio repentino al entrenar o competir

Inicio gradual en varios entrenamientos consecutivos o competencias sin una única causa que lo provocase

1. ¿Durante cuánto tiempo esa lesión limitó su entrenamiento normal?

1-7 días

8-21 días

Más de 21 días

1. ¿Consultó a un médico o fisioterapeuta deportivo por esa dolencia?

Sí

No Si no lo hizo, ¿por qué?

Preferí gestionar el problema por mi cuenta

Mi entrenador pudo ocuparse del problema

No tenía asistencia médica en ese momento

Otro

1. ¿Ha sufrido alguna **otra lesión** (no relacionada con el deporte) durante **los últimos 12 meses**?

Sí

No (🡪 pregunta n.° 16)

1. ¿Qué causó la lesión?

Un accidente, por ejemplo, de tráfico.

Violencia interpersonal

Otro

1. ¿Durante cuánto tiempo esa lesión limitó su entrenamiento normal?

1-7 días

8-21 días

Más de 21 días

1. ¿Consultó a un médico u otro profesional médico sobre la lesión?

Sí

No Si no lo hizo, ¿por qué?

Preferí gestionar el problema por mi cuenta

Mi entrenador pudo ocuparse del problema

No tenía asistencia médica en ese momento

Otro

1. **1. Sus experiencias de acoso y abuso físico**
2. ¿Un adulto le ha hecho alguna de las siguientes cosas y, de ser así, en qué **contexto** y **con qué frecuencia**?

***En el Atletismo Fuera del Atletismo***

Nunca A veces A menudo Nunca A veces A menudo

Lo insultó

Lo obligó a entrenar contra su voluntad

Lo amenazó con golpearlo

Lo aisló de sus amigos

Lo apartó, empujó o sacudió

Le arrojó algo

Le causó dolor físico o daño

Le hizo daño con las manos

Le dio patadas, mordiscos o puñetazos

Lo atacó físicamente de otra manera

Lo amenazó con dañarlo o dañó

a un ser querido

Si todas las respuestas son negativas, 🡪 pregunta n.° 20.

1. ¿Cuántos años tenía la primera vez que sucedió? Años
2. ¿Quién se lo hizo?

*Puede marcar varias respuestas.*  Padre (padre/madre biológicos, padrastro/madrastra)

Hermanos (biológicos/hermanastros)

Otro pariente

Amigo o conocido

Su pareja (novio/novia)

Otro atleta

Entrenador atlético, entrenador, personal médico

Profesor

Alguien totalmente desconocido

1. ¿Consultó a un médico o terapeuta como reacción a lo que le sucedió?

Sí

No, no había motivos

No, pero ahora creo que debería haberlo hecho

**D.2. Sus experiencias de abuso sexual**

1. ¿**Alguna vez** lo han convencido, presionado o forzado a realizar actos sexuales en contra de su voluntad en su vida **fuera del atletismo**?

*Puede marcar varias respuestas.*

No he sufrido lo anterior en contra de mi voluntad (🡪 Fin de la encuesta)

Alguien se expuso ante usted

Alguien le tocó los genitales o intentó desnudarlo para mantener relaciones sexuales con usted

Ha masturbado a alguien

Ha tenido relaciones sexuales vaginales

Ha practicado sexo oral

Ha practicado sexo anal

1. ¿Cuántas veces pasó?  Una vez

2-5 veces

Más de 5 veces

1. ¿Qué edad tenía la primera vez que sufrió abuso sexual? Años
2. ¿Alguna vez lo han convencido, presionado o forzado a realizar actos sexuales contra su voluntad **en relación con actividades o reuniones de atletismo**?

*Puede marcar varias respuestas.*

No he sufrido lo anterior en contra de mi voluntad (🡪 Fin de la encuesta)

Alguien se expuso ante usted

Alguien le tocó los genitales o intentó desnudarlo para mantener relaciones sexuales con usted

Ha masturbado a alguien

Ha tenido relaciones sexuales vaginales

Ha practicado sexo oral

Ha practicado sexo anal

1. ¿Qué edad tenía la primera vez que sufrió abuso sexual? Años
2. ¿Quién se lo hizo?

*Puede marcar varias respuestas.*  Padre (padre/madre biológicos, padrastro/madrastra)

Hermanos (biológicos/hermanastros)

Otro pariente

Amigo o conocido

Su pareja (novio/novia)

Otro atleta

Entrenador atlético, entrenador, personal médico

Profesor

Alguien totalmente desconocido

1. ¿Habló con un médico o autoridades competentes en relación con lo sucedido?

Sí

No, no había motivos

No, pero ahora creo que debería haberlo hecho

1. ¿Estaba borracho o drogado la primera vez que sucedió en el contexto de actividades o reuniones de atletismo?  Sí

No

1. ¿Qué formas de persuasión, presión o fuerza utilizó la persona en cuestión en relación con actividades o reuniones atléticas? *Puede marcar varias respuestas.*

Lo engañó

Abusó de su posición

Le convenció

Le amenazó con rechazarlo

Le agarró

Le golpeó o hizo daño

Le proporcionó alcohol, drogas o píldoras

Otro

1. ¿Intentó la persona en cuestión compensarlo con regalos, dinero, etc.?

Sí

No

1. ¿Alguna vez buscó ayuda o apoyo en relación con lo siguiente?

Sí No

Ser víctima de abuso psicológico

Ser víctima de abuso físico

Ser víctima de abuso sexual

Denunciar a alguien por cometer abuso sexual

Tener problemas con los padres

Sufrir problemas de salud mental

Otro

1. ¿A quién pidió ayuda?

*Puede marcar varias respuestas.*  Padres

Hermanos

Novia/novio

Amigo de la misma edad

Pariente o amigo adulto

«Profesionales»: profesores, terapeutas, apoyo social, enfermeros o equivalente

«Trabajador de atletismo»: entrenadores, personal del club o equivalente

Otra persona

Se denunció a los servicios sociales o a la policía

1. ¿Recibió el apoyo y la ayuda que necesitaba?

Sí

No

1. Si ha denunciado episodios de acoso y/o abuso, ¿está satisfecho con cómo se gestionó?

Sí

No

1. ¿Conoce alguna política de amparo o código de conducta que haya implantado su Federación Nacional?

Sí

No

Envíe sus datos pulsando el botón Enviar.
