# Supplementary material for: Lifetime Prevalence of Verbal, Physical, and Sexual Abuses in Young Elite Athletics Athletes
Source: Front Sports Act Living. 2021 May 31;3:657624. doi: 10.3389/fspor.2021.657624 (PMC8200562; doi:10.3389/fspor.2021.657624)
Supplement: Supplementary file 10 [file Table_10.DOCX]

**健康、幸福感及受骚扰和侵犯经历问卷**

调查问卷由四部分组成，完成问卷需要约5-6分钟：

A - 个人信息（1分钟）

B - 您的幸福感（1分钟）

C - 您的健康状况（1分钟）

D1，D2 – 受骚扰和侵犯的经历（3分钟）

回答调查时请认真考虑以下关键词的定义：

**骚扰**

骚扰指不必要的关注或行为，侵犯尊严和/或构成带威胁、敌意、恐吓、侮辱人格或攻击性的环境。

**侵犯**

侵犯指某人的权利受到他人的侵犯。属于滥用权力和信任的侵犯。

**个人信息**

1. 您多少岁？ 年龄
2. 性别  女性

男性

1. 您来自哪个地区？ 北美洲

中美洲和加勒比群岛地区

南美洲

欧洲

东欧和高加索

北非

中部非洲

南部非洲

中东

中亚

南亚

东亚

东南亚

大洋洲

1. 您多少岁开始田径运动？  < 8岁  8-12 岁  > 12 岁
2. 您的主赛事属于哪一组比赛？ 跳跃

投掷

短跑

中长跑

全能运动

竞走

1. 您平均每周花几个小时训练和/或参加田径比赛？

小时数

1. **您的幸福感**
2. 请查看以下五种描述，选择与您**最近两周**感觉最接近的一项。注意数字越高意味着幸福感越强。例如：如果在过去的两周内您有一半以上的时候都感觉很快乐，精神状态很棒的话，请在右上角的数字3处打勾。

|  | 近两周 | 始终 | 多数时间 | 一半以上的时间 | 少于一半的时间 | 偶尔 | 完全没有 |
| --- | --- | --- | --- | --- | --- | --- | --- |
| **1** | 我感觉很快乐，精神状态很棒 | 5 | 4 | 3 | 2 | 1 | 0 |
| **2** | 我感到平静和放松 | 5 | 4 | 3 | 2 | 1 | 0 |
| **3** | 我感到活跃，充满活力 | 5 | 4 | 3 | 2 | 1 | 0 |
| **4** | 我醒来时感觉很清新，精力充沛 | 5 | 4 | 3 | 2 | 1 | 0 |
| **5** | 我的日常生活充满了令我感兴趣的事情 | 5 | 4 | 3 | 2 | 1 | 0 |

1. **您的健康状况**
2. 在**过去的12个月**里，您是否因**运动相关伤痛**而限制了您的正常训练？

是

否 （🡪12题）

1. 伤痛最初是怎么发生的？

创伤事件之后，例如碰撞/摔倒

训练或竞赛时突然发作

无单一诱因，连续多次训练或比赛导致的逐渐发作

1. 该伤痛限制了您多久的正常训练？

1-7天

8-21天

大于 21天

1. 您是否就此疾病咨询了运动医师或理疗师？

是

否 如果没有，为什么？

我更希望自己处理

我的教练可以应对

我当时没有任何医疗支持

其他

1. 您在**过去的12个月**中是否受过**其他伤痛**（与体育无关）？

是

否（🡪 16题）

1. 伤痛的原因是什么？

意外，例如交通事故.

人际暴力冲突

其他

1. 该伤痛限制了您多久的正常训练时间？

1-7天

8-21天

大于21天

1. 您是否曾就此伤痛咨询医师或其他医疗专业人员？

是

否 如果没有，为什么？

我更希望自己处理

我的教练可以应对

我当时没有任何医疗支持

其他

1. **1. 您受到骚扰和身体侵犯的经历**
2. 是否有成年人对您做过以下事情？如果有，是在什么**情况**下？**多久会发生一次**？

**田径运动内**  **田径运动外**

从未 偶尔 经常 从未 偶尔 经常

辱骂

违背您的意愿逼迫训练

威胁要打您

在朋友中孤立您

推、撞或摇动您

朝您扔东西

对您的身体造成疼痛或伤害

用手伤害您

拳打脚踢或咬您

用其他方式对您人身攻击

威胁伤害或伤害您珍视的人

如果所有回答都为否🡪 20题

1. 首次发生时您几岁？ 岁
2. 谁是施害者？

可多选父母（亲生父亲/母亲，继父/继母）

兄弟姐妹（亲生/异父母的兄弟姐妹）

其他亲属

朋友或认识的人

您的伴侣（男朋友/女朋友）

其他运动员

运动训练师、教练、医务人员

教师

完全不认识的人

1. 您是否曾就发生在您身上的事咨询医师或心理辅导员？

是

否，没有理由

否，但我现在认为我当时应该咨询

**D.2. 您的性侵经历**

1. **在田径运动之外**，您是否**曾**被人说服、鼓动或被迫发生性行为（非本人意愿）？

可多选

我未承受过上述违背我意愿的行为（🡪结束调查问卷）

有人（男/女）在您面前露体

有人触摸了您的生殖器或试图脱下您的衣服发生性关系

你自慰某人

您有过阴道性交

您有过口交

您有过肛交

1. 这种行为发生过多少次？  一次

2-5 次

多于5次

1. 您首次遭遇性侵时多少岁？ 岁
2. **在体育活动或聚会上，**您有没有被人说服、鼓动或被迫发生性行为（非本人意愿）？

可多选

我未承受过上述违背我意愿的行为（🡪结束调查问卷）

有人（男/女）在您面前露体

有人触摸了您的生殖器或试图脱下您的衣服并发生性关系

你自慰某人

您有过阴道性交

您有过口交

您有过肛交

1. 您首次遭遇性侵时多少岁？ 岁
2. 谁是施害者？

可多选父母（亲生父亲/母亲，继父/继母）

兄弟姐妹（亲生/异父母的兄弟姐妹）

其他亲属

朋友或认识的人

您的伴侣（男朋友/女朋友）

其他运动员

运动训练师、教练、医务人员

教师

完全不认识的人

1. 您是否曾就发生在您身上的事咨询医师或相关部门？

是

否，没有理由

否，但我现在认为我当时应该咨询

1. 在体育活动或聚会上首次发生这种事时您是否喝醉或被下药？

是

否

1. 在体育活动或聚会上，施害者采用什么形式说服、施压或强迫您？可多选

欺骗您

滥用职权

劝服您

威胁开除您

强迫您

打您或伤害您

提供酒精或药物

其他

1. 施害者是否尝试用礼物、钱等形式补偿您？

是

否

1. 您是否曾就以下方面寻求过帮助或支持：

是 否

作为心理侵害的受害者

作为身体侵害的受害者

作为性侵害的受害者

举报某人实施性侵犯

与父母有矛盾

有心理健康问题

其他

1. 您向谁寻求帮助？

可多选  父母

兄弟姐妹

女朋友/男朋友

同龄朋友

成年亲属或朋友

“专业人士” - 教师、辅导员、社会工作者、护士或

同等资质人士

“田径官员” - 教练、俱乐部官员或同等资质人士

其他人士

上报至社会服务部门或警察

1. 您是否获取您需要的支持和帮助？

是

否

1. 如果您报告过骚扰和/或侵犯事件，您是否对这件事的处理方式感到满意？

是

否

1. 您是否知道您所在国的全国联合会就此实施何种保障政策或行为准则？

是

否

按下提交键发送您的数据。
