# Supplementary material for: Lifetime Prevalence of Verbal, Physical, and Sexual Abuses in Young Elite Athletics Athletes
Source: Front Sports Act Living. 2021 May 31;3:657624. doi: 10.3389/fspor.2021.657624 (PMC8200562; doi:10.3389/fspor.2021.657624)
Supplement: Supplementary file 11 [file Table_11.DOCX]

**Questionário sobre bem-estar, saúde e experiências de assédio e abuso**

O inquérito consiste 4 secções e leva cerca de 5-6 minutos a completar:

A – Informação pessoal (1 min)

B – O teu bem-estar (1 min)

C – A tua saúde (1 min)

D 1, D2 – experiências de assédio e abuso (3 mins)

Por favor considere as seguintes definições chaves quando responder ao inquérito:

**Assédio**

Assédio está relacionado com atenção ou conduta não desejada, violação da dignidade e/ou criação de um ambiente ameaçador, hostil, intimidante, degradante, humilhante ou ofensivo.

**Abuso**

Abuso implica que os direitos da pessoa foram violados por outro. Isto é baseado em abuso de poder ou de confiança.

**informação pessoal**

1. Quantos anos tens? Idade
2. Sexo  Feminino

Masculino

1. De que área geográfica és oriundo?  América do Norte

América Central e Caraíbas

América do Sul

Europa

Europa de Leste e Cáucaso

Norte de África

África Central

África Austral

Médio Oriente

Ásia Central

Ásia do Sul

Este da Ásia

Sudeste Asiático

Oceânia

1. Com que idade começaste o atletismo?  < 8 anos  8-12 anos  > 12anos
2. A que grupo de eventos pretence o teu evento principal?

Saltos

Lançamentos

velocidade

Meio-fundo/fundo

Provas Combinadas

Marcha atlética

1. Em media, quantas horas por semana despendes em treino ou competição?

Horas

1. **Acerca do teu Bem-estar**
2. Por favor indica para cada uma das cinco afirmações seguintes, o que é mais aproximado sobre a forma como te tens sentido nas passadas duas semanas. Toma em atenção que o número mais alto quer dizer melhor bem-estar. Por exemplo: Exemplo: Se sentiste-te alegre e bem disposto(a) mais de metade do tempo durante as passadas duas semanas, coloca uma cruz na caixa assinalada com o número 3.

|  | Durante as últimas duas semanas | Todo o tempo | A maior parte do tempo | Mais de metade do tempo | Menos de metade do tempo | Algumas  vezes | Nunca |
| --- | --- | --- | --- | --- | --- | --- | --- |
| **1** | **Senti-me alegre e bem disposto(a)** | 5 | 4 | 3 | 2 | 1 | 0 |
| **2** | **Senti-me calmo(a) e tranquilo(a)** | 5 | 4 | 3 | 2 | 1 | 0 |
| **3** | **Senti-me activo(a) e enérgico(a)** | 5 | 4 | 3 | 2 | 1 | 0 |
| **4** | **Acordei a sentir-me fresco(a) e repousado(a)** | 5 | 4 | 3 | 2 | 1 | 0 |
| **5** | **O meu dia-a-dia tem sido preenchido com coisas que me interessam** | 5 | 4 | 3 | 2 | 1 | 0 |

1. **Acerca da tua saúde**
2. Nos últimos 12 meses sofreu alguma lesão relacionada com o desporto que restringiu o treino habitual?

Sim

Não (🡪 questão n. 12)

1. Como aconteceu inicialmente a lesão?

Como consequência de um episódio traumático, por exemplo colisão e queda

Início súbito enquanto treinava e competia

Início gradual ao longo de sessões de treino consecutivas ou competição sem um evento causal isolado.

1. Quanto tempo a lesão restringiu o treino habitual?

1-7 dias

8-21 dias

Mais do que 21 dias

1. Consultaste algum especialista em Medicina Desportiva ou fisioterapeuta por causa das tuas queixas?

Sim

Não. Se não, porquê?

Preferi tratar pessoalmente do assunto

O meu treinador conseguiu tratar do problema

Não tinha apoio médico na altura

Outra

1. Nos últimos 12 meses sofreste outra qualquer lesão (não relacionada com o desporto)?

Sim

Não (🡪 questão n. 16)

1. o que ocasionou a lesão?

Um acidente, por exemplo de trânsito

Violência interpessoal

Outra

1. Quanto tempo a lesão restringiu o treino habitual?

1-7 dias

8-21 dias

Mais do que 21 dias

1. Consultaste algum medico ou outra profissional de saúde por causa da lesão?

sim

Não Se não, porquê?

Preferi tratar pessoalmente do assunto

O meu treinador conseguiu tratar do problema

Não tinha apoio médico na altura

Outra

1. **1. As tuas experiências de assédio ou abuso físico**
2. Aconteceu que um adulto fez alguma coisa a ti, e, em caso afirmativo, em que contexto e com que frequência?

***No atletismo***  ***Fora do atletismo***

Nunca Algumas vezes Frequentemente Nunca Algumas vezes frequentemente

Insultou

Obrigou a treinar contra a tua vontade

Ameaçou bater-te

isolou-te dos teus amigos

Empurrou ou chocou-te

Atirou algo contra ti

Causou-lhe dor física ou dano

Magoou-te com as suas mãos

Pontapeou-te, mordeu-te ou bateu-te com os seus punhos

Fisicamente atacou-te de alguma outra forma

Ameaçou magoar-te ou magoou alguém

querido para ti

Se todas as questões forem negativas 🡪 questão n. 20.

1. Com que idade é aconteceu pela primeira vez? Anos
2. Quem te fez isto?

*Podem ser assinaladas várias opções.* Pais (pai/mãe biológico, padrasto/madrasta)

Irmãos (biológicos/meio-irmãos)

Outros parentes

Amigos ou conhecidos

Teu parceiro(a) (namorado/namorada)

Outro atleta

Treinador(a) ou pessoal médico

Professor(a)

Desconhecido(a)

1. Consultaste um médico ou conselheiro relativamente ao que te tinha acontecido?

Sim

Não, não havia razão

Não, mas agora acredito que deveria ter feito

**D.2. As tuas experiências de abuso sexual**

1. Já foste persuadido, empurrado a ou forçado a acto sexuais contra a tua vontade fora do atletismo ou no atletismo?

*Podem ser assinaladas várias opções.*

Nunca fui sujeito(a) a tal contra minha vontade (🡪 fim do questionário)

Alguém se exibiu nú a ti

Alguém tocou os teus genitais ou tentou despir-te, ter sexo contigo

Masturbou alguém

Tiveste relação sexual vaginal

Tiveste sexo oral

Tiveste sexo anal

1. Quantas vezes aconteceu?  Uma vez

2-5 vezes

Mais do que 5 vezes

1. Com que idade experimentaste pela primeira vez o abuso sexual? Years

anos

1. Já foste persuadido, empurrado a ou forçado a acto sexuais contra a tua vontade, **relacionado com actividades atleticas ou encontros**?

*Podem ser assinaladas várias opções.*

Nunca fui sujeito(a) a tal contra minha vontade (🡪 fim do questionário)

Alguém se exibiu nú a ti

Alguém tocou os teus genitais ou tentou despir-te, ter sexo contigo

Masturbou alguém

Tiveste relação sexual vaginal

Tiveste sexo oral

1. Com que idade experimentaste pela primeira vez o abuso sexual? anos
2. Quem te abusou?

*Podem ser assinaladas várias opções.*

Pais (pai/mãe biológico, padrasto/madrasta)

Irmãos (biológicos/meio-irmãos)

Outros parentes

Amigos ou conhecidos

Teu parceiro(a) (namorado/namorada)

Outro atleta

Treinador(a) ou pessoal médico

Professor(a)

Desconhecido(a)

1. Consultaste algum medico ou a autoridade em resposta ao que te tinha acontecido?

Sim

Não, não havia razão

Não, mas agora acredito que deveria ter feito

1. Estavas bebado(a) ou drogado(a) a primeira vez que aconteceu no contexto de actividades atléticas ou encontros?  sim

não

1. Que formas de persuasão, pressão ou forçar a pessoa em questao usou, em relação com as actividades atléticas ou encontros. *Podem ser assinaladas várias opções*.

Enganou-te

Abusou da sua posição

Persuadiu-te

Ameaçou rejeitar-te

Segurou-te

Bateu-te ou magoou-te

Deu-te álcool, drogas ou comprimidos

Outras

1. A pessoa em questão tentou compensar-te com presentes ou dinheiro, por exemplo?

sim

não

1. Algumas vezes procurou ajuda ou apoio em relação a:

sim não

Ter sido vítima de abuso psicológico

Ter sido vítima de abuso físico

Ter sido vítima de abuso sexual

Denunciou alguém por abuso sexual

ter problemas com os pais

Vivenciar problemas de saúde mental

Outros

1. A quem pediu ajuda?

*Podem ser assinaladas várias opções*  Pais

Irmãos

namorada/namorado

Amigo da mesma idade

Parente ou amigo adulto

"Profissional" – professor(a), conselheiro, assistente social, enfermeiro ou equivalente

"Oficial de atletismo" - treinador, oficial do clube, ou equivalente

Outra pessoa

Foi denunciado aos serviços sociais ou à polícia

1. Recebeste o apoio e a ajuda que necessitavas?

sim

não

1. Se alguma vez denunciaste episódios de assédio e/ou abuso, ficaste satisfeito com a forma como foi lidado?

sim

não

1. Tens conhecimento de alguma política de salvaguarda ou código de conduta implementado pela tua federação nacional?

sim

não

Envia os teus dados carregando no botão de submeter
