# Supplementary material for: Lifetime Prevalence of Verbal, Physical, and Sexual Abuses in Young Elite Athletics Athletes
Source: Front Sports Act Living. 2021 May 31;3:657624. doi: 10.3389/fspor.2021.657624 (PMC8200562; doi:10.3389/fspor.2021.657624)
Supplement: Supplementary file 12 [file Table_12.DOCX]

**Questionnaire on** **wellbeing, health, and experiences of harassment and abuse**

The survey consists of four sections and takes approximately 5-6 minutes to complete:

A - Personal information (1 min)

B - Your wellbeing (1 min)

C - Your health (1 min)

D 1, D2 - Experiences of harassment and abuse (3 mins)

Please consider following key definitions when you answer the survey:

**Harassment**

Harassment relates to unwanted attention or conduct, the violation of dignity and/or the creation of a threatening, hostile, intimidating, degrading, humiliating or offensive environment.

**Abuse**

Abuse implies that a person’s rights are violated by another. This is based on the abuse of power and trust.

**Personal information**

1. How old are you? Age
2. Sex  Female

Male

1. Which geographical area are you from?  North America

Central America and Caribbean islands

South America

Europe

Eastern Europe and Caucasus

Northern Africa

Central Africa

Southern Africa

Middle East

Central Asia

Southern Asia

Eastern Asia

South-East Asia

Oceania

1. How old were you when you started athletics?  < 8 years  8-12 years  > 12 years
2. To what group events does your main event belong?  Jumps

Throws

Sprints

Middle/long-distance running

Combined events

Race walk

1. How many hours on average do you spend training and/or competing in athletics per week?

Hours

1. **About your wellbeing**
2. Please indicate for each of the five statements, which is closest to how you have been feeling **over the last two weeks.** Notice that higher numbers mean better well-being. Example: If you have felt cheerful and in good spirits more than half of the time during the last two weeks, put a tick in the box with the number 3 in the upper right corner.

|  | Over the last two weeks | All of the time | Most of the time | More than half of the time | Less than half of the time | Some of the time | At no time |
| --- | --- | --- | --- | --- | --- | --- | --- |
| **1** | **I have felt cheerful and in good spirits** | 5 | 4 | 3 | 2 | 1 | 0 |
| **2** | **I have felt calm and relaxed** | 5 | 4 | 3 | 2 | 1 | 0 |
| **3** | **I have felt active and vigorous** | 5 | 4 | 3 | 2 | 1 | 0 |
| **4** | **I woke up feeling fresh and rested** | 5 | 4 | 3 | 2 | 1 | 0 |
| **5** | **My daily life has been filled with things that interest me** | 5 | 4 | 3 | 2 | 1 | 0 |

1. **About your health**
2. Have you during **the past 12 months** suffered any **sports-related injury** that restricted your normal training?

Yes

No (🡪 question n. 12)

1. How did the injury first occur?

Following a traumatic event, e.g. collision/fall

Sudden onset while training or competing

Gradual onset over several consecutive trainings or competition with no single causative event

1. How long did the injury restrict your normal training?

1-7 days

8-21 days

Longer than 21 days

1. Did you consult a sports physician or physiotherapist for the complaint?

Yes

No If not, why?

I preferred managing the issue on my own

My coach could handle the problem

I didn’t have any medical support at that time

Other

1. Have you during **the past 12 months** suffered any **other injury** (unrelated to sports)?

Yes

No (🡪 question n. 16)

1. What caused the injury?

An accident, e.g. in traffic.

Inter-personal violence

Other

1. How long did the injury restrict your normal training?

1-7 days

8-21 days

Longer than 21 days

1. Did you consult a physician or other medical professional for the injury?

Yes

No If not, why?

I preferred managing the issue on my own

My coach could handle the problem

I didn’t have any medical support at that time

Other

1. **1. Your experiences of harassment and physical abuse**
2. Has it happened that an adult did any of the following to you, and if so, in what **context and how often**?

***Inside Athletics***  ***Outside Athletics***

Never Sometimes Often Never Sometimes Often

Insulted you

Obliged you to train against your will

Threatened to hit you

Isolated you from friends

Pushed, shoved or shook you

Threw something at you

Caused you physical pain or harm

Hurt you with his/her hands

Kicked, bit or hit you with his/her fists

Physically attacked you otherwise

Threatened to harm or harmed

someone dear to you

If all answers are negative 🡪 question n. 20.

1. How old were you the first time it happened? Years
2. Who did that to you?

*Several answers may be marked.* Parent (Biological father/mother, stepfather/stepmother)

Siblings (biological/step-siblings)

Other relative

Friend or acquaintance to you

Your partner (boyfriend/girlfriend)

Other athlete

Athletic trainer, Coach, Medical staff

Teacher

Someone totally unknown

1. Did you see a physician or counselor in response to what has happened to you?

Yes

No, there was no reason

No, but I now believe that I should have

**D.2. Your experiences of sexual abuse**

1. Have you **ever** been persuaded, pushed or forced into sexual acts against your will in your life, **outside of athletics**?

*Several answers may be marked*

I have not been subjected to the above against my will (🡪 question n.23)

Someone has exposed himself/herself to you

Someone has touched your genitals or tried to undress you, to have sex with you

You have masturbated for someone

You have had vaginal intercourse

You have had oral sex

You have had anal sex

1. How many times did that happen?  Once

2-5 times

More than 5 times

1. How old were you the first time you experienced sexual abuse? Years
2. Have you ever been persuaded, pushed or forced into sexual acts against your will, **in connection with athletic activities or gatherings**?

*Several answers may be marked*

I have not been subjected to the above against my will (🡪 End survey)

Someone has exposed himself to you

Someone has touched your genitals or tried to undress you, to have sex with you

You have masturbated for someone

You have had vaginal intercourse

You have had oral sex

You have had anal sex

1. How old were you the first time you experienced sexual abuse? Years
2. Who did that to you?

*Several answers may be marked.* Parent (Biological father/mother, stepfather/stepmother)

Siblings (biological/step-siblings)

Other relative

Friend or acquaintance to you

Your partner (boyfriend/girlfriend)

Other athlete

Athletic Trainer, Coach, Medical staff

Teacher

Someone totally unknown

1. Did you see a physician or relevant authority in response to what has happened to you?

Yes

No, there was no reason

No, but I now believe that I should have

1. Were you drunk or drugged the first time it happened in the context of athletics activities or gatherings?  Yes

No

1. What forms of persuasion, pressure or force, did the person in question, use in connection with athletic activities or gatherings? *Several answers may be marked*.

Fooled you

Abused his position

Persuaded you

Threatened to reject you

Held you

Hit you or hurt you

Provided alcohol, drugs or tablets

Other

1. Did the person in question try to compensate you in the form of gifts, money, etc.?

Yes

No

1. Have you ever sought help or support in respect to:

Yes No

Being a victim of psychological abuse

Being a victim of physical abuse

Being a victim of sexual abuse

Reporting someone for committing sexual abuse

Having problems with parents

Experiencing mental health problems

Other

1. Whom did you ask for help?

*Several answers may be marked*  Parents

Siblings

Girlfriend / Boyfriend

Same aged friend

Adult relative or friend

"Professional" - teachers, counselors, social support, nurse or equivalent

"Athletics official" - coaches, club official, or equivalent

Other person

It was reported to social services or the police

1. Did you receive the support and the help that you needed?

Yes

No

1. If you have reported episodes of harassment and/or abuse, are you satisfied with the way this was dealt with?

Yes

No

1. Are you aware of any safeguarding policy or code of conduct implemented by your National Federation?

Yes

No

Send your data by pressing the Submit button.
