# Supplementary material for: Lifetime Prevalence of Verbal, Physical, and Sexual Abuses in Young Elite Athletics Athletes
Source: Front Sports Act Living. 2021 May 31;3:657624. doi: 10.3389/fspor.2021.657624 (PMC8200562; doi:10.3389/fspor.2021.657624)
Supplement: Supplementary file 13 [file Table_13.DOCX]

**استبيان حول الرفاهية والصحة وتجارب التحرّش والإساءة**

يتكوّن الاستقصاء من أربعة أقسام ويستغرق استكماله ما يقرب من 5الى 6 دقائق**:**

أ - المعلومات الشخصية (دقيقة واحدة)

ب - رفاهيتك (1 دقيقة)

ج - صحتك (1 دقيقة)

د1، د2- تجارب التحرّش والإساءة (3 دقائق)

يرجى (النظر ف اتباع التعريفات الأساسية التالية عند الإجابة على الاستبيان**:**

**التحرّش**

يتعلّق التحرش بسلوك أو اهتمام غير مرغوب فيه، وانتهاك للكرامة و/أو خلق بيئة مهدّدة أو معادية أو مخيفة أو مذلّة أو مهينة أو عدائية**.**

**الإساءة**

تعني الإساءة أن شخصاً ما ينتهك حقوق شخص آخر. ويرتكز ذلك على إساءة استخدام السلطة والثقة**.**

**المعلومات الشخصية**

1. كم عمرك؟ السنّ
2. الجنس أنثى

ذكر

1. من أي منطقة جغرافية أنت؟ أميركا الشمالية

أمريكا الوسطى وجزر الكاريبي

أميركا الجنوبية

أوروبا

شرق أوروبا والقوقاز

شمال أفريقيا

أفريقيا الوسطى

جنوب أفريقيا

الشرق الأوسط

آسيا الوسطى

جنوب آسيا

شرق آسيا

جنوب شرق آسيا

أوقيانيا

1. كم كان عمرك عندما بدأت ألعاب القوى؟ > 8 سنوات 8-12 سنة < 12 سنة
2. إلى أي مجموعة فعاليات ينتمي إليهـا تخصصگ الرئيسي**?**

القفز

الرمي

سباق السرعة

الجري على مسافة متوسطة / طويلة

أحداث مجتمعة

سباق

1. كم عدد الساعات التي تقضيها في المتوسط في التدريب و/ أو التنافس في ألعاب القوى في الأسبوع؟

الساعات

1. **رفاهيّتك**
2. يرجى الإشارة إلى كأي عبارة، بين العبارات الخمسة، هي الأقرب إلى ما كنت تشعر به **خلال الأسبوعين الماضيين**. لاحظ أن الأرقام المرتفعة تعني رفاهية أفضل. مثال: إذا كنت شعرت بالبهجة وفي حالة معنوية جيدة أكثر من نصف الوقت خلال الأسبوعين الماضيين، ضع علامة في المربع مع الرقم 3 في الزاوية اليمنى العليا.

|  | خلال الأسبوعين الأخيرين | طوال الوقت | معظم الوقت | أكثر من نصف الوقت | أقل من نصف الوقت | بعض الوقت | ولا مرة |
| --- | --- | --- | --- | --- | --- | --- | --- |
| **1** | **شعرت بالبهجة وفي حالة معنوية جيدة** | 5 | 4 | 3 | 2 | 1 | 0 |
| **2** | **شعرت بالهدوء والاسترخاء** | 5 | 4 | 3 | 2 | 1 | 0 |
| **3** | **شعرت بنشاط وحيوية** | 5 | 4 | 3 | 2 | 1 | 0 |
| **4** | **استيقظت مرتاحاً** | 5 | 4 | 3 | 2 | 1 | 0 |
| **5** | **امتلأت حياتي اليومية بالأشياء التي تهمني** | 5 | 4 | 3 | 2 | 1 | 0 |

1. **صحتك**
2. هل عانيت **خلال الأشهر الـ 12 الماضية** من أي **إصابات مرتبطة بالرياضة** تقيّد تدريبك العادي**؟**

نعم

كلا (←السؤال رقم 12)

1. كيف حدثت الإصابة لأول مرة؟

بعد حادث صادم، على سبيل المثال تصادم/ سقوط

ظهور مفاجئ أثناء التدريب أو التنافس

بداية تدريجية على عدة تدريبيات متتالية أو منافسة بدون أي مسبّب واحد

1. إلى متى أدت الإصابة إلى تعطيل تدريبك العادي؟

1-7 أيام

8-21 يوماً

أطول من 21 يوماً

1. هل استشرت طبيبًا رياضيًا أو أخصائياً للعلاج الطبيعي؟

نعم

كلا إذا لا، لماذا؟

فضلت إدارة المشكلة بمفردي

تمكّن لمدربى التعامل مع المشكلة

لم يكن لدي أي دعم طبي في ذلك الوقت

أسباب أخرى

1. هل تعرضت **خلال الأشهر الـ 12 الماضية** لأي **إصابة أخرى** (لا علاقة لها بالرياضة)؟

نعم

كلا (**←**  السؤال رقم 16)

1. ما سبّب الإصابة؟

حادث، على سبيل المثال حادث سير**.**

عنف بين الأفراد

أسباب أخرى

1. إلى متى أدت الإصابة إلى تعطيل تدريبك العادي؟

1-7 ايام

8-21 يوماً

أطول من 21 يوماً

1. هل قمت باستشارة طبيب أو أخصائي طبي آخر للإصابة؟

نعم

كلا إذا لا، لماذا؟

فضّلت إدارة المشكلة بمفردي

تمكّن مدربى التعامل مع المشكلة

لم يكن لدي أي دعم طبي في ذلك الوقت

أسباب أخرى

**D.1** **– تجاربك في التحرش والإساءة الجسدية**

1. هل حدث أن شخصًا بالغًا قام بأحد الأمور التالية لك، وإذا كان الأمر كذلك، في **أي سياق وكم مرة**؟

**داخل ألعاب القوى خارج ألعاب القوى**

أبداً أحياناً غالباً أبداً أحياناً غالباً

أهانك

أجبرك على التدريب ضد إرادتك

هدّد بيضربك

عزلك عن الأصدقاء

دفعك أو بطحك أو هزك

رمى شيئاً عليك

تسبب لك بالألم الجسدي أو الأذى

آذاك بيديه

ركلك، عضك أو ضربك بقبضة يده

هاجمك جسدياً خلاف ذلك

هدد بالضرر أو أضر شخص عزيز عليك

إذا كانت جميع الإجابات سلبية ←السؤال رقم. 20.

17) كم كان عمرك في المرة الأولى التي حدث فيها ذلك؟ السنوات

**18) من** فعل ذلك بك؟

يمكن وضع علامة على عدة إجابات*.*

الأهل البيولوجيون (الأب/ الأم، زوج الأم / زوجة الأب)

الأشقاء (البيولوجيون / الأخوة من غير الأشقاء)

أقرباء آخرون

صديق أو أحد معارفك

شريك حياتك (صديقك الحميم / صديقتك الحميمة)

رياضي آخر

مدرب رياضي، مدرب، طاقم طبي

مدرّس

شخص لا تعرفه إطلاقاً

19) هل استشرت طبيباً أو المستشاراً رداً على ما حدث لك؟

نعم

لا، لا داعي لذلك

لا، لكنني أعتقد الآن أنه كان يجدر بي فعل ذلك

**D.2** **تجاربك في الإساءة الجنسية**

20) هل تم إقناعك أو دفعك أو إجبارك **يوماُ** على القيام بأعمال جنسية ضد إرادتك في حياتك، **خارج ألعاب القوى**؟

يمكن وضع علامة على عدة إجابات

لم أتعرض لذلك ضد إرادتي ← نهاية المسح)

شخص تعرّى أمامك

شخص لمس أعضائك التناسلية أو حاول خلع ملابسك، أو ممارسة الجنس معك

لقد استمنيت على شخص ما

مارست الجنس

مارست الجنس عن طريق الفم

مارست الجنس الشرجي

21) كم مرة حدث ذلك؟ مرة

2-5 مرات

أكثر من 5 مرات

22) كم كان عمرك في المرة الأولى التي عانيت فيها من الإساءة الجنسية؟ السنوات

23) هل تم إقناعك أو إجبارك أو إرغامك على القيام بأعمال جنسية ضد إرادتك، **في ما له صلة بالأنشطة أو الفعاليات الرياضية؟**

يمكن وضع علامة على عدة إجابات

لم أتعرض لذلك ضد إرادتي ← نهاية المسح)

شخص تعرّى أمامك

شخص لمس أعضائك التناسلية أو حاول خلع ملابسك، أو ممارسة الجنس معك

لقد استمنيت على شخص ما

مارست الجنس

مارست الجنس عن طريق الفم

مارست الجنس الشرجي

24) كم كان عمرك في المرة الأولى التي عانيت فيها من الإساءة الجنسية؟ السنوات

25) من فعل هذا بك؟

يمكن وضع علامة على عدة إجابات*.*

الأهل البيولوجيون (الأب/ الأم، زوج الأم / زوجة الأب)

الأشقاء (البيولوجيون / الأخوة من غير الأشقاء)

أقرباء آخرون

صديق أو أحد معارفك

شريك حياتك (صديقك الحميم / صديقتك الحميمة)

رياضي آخر

مدرب رياضي، مدرب، طاقم طبي

مدرّس

شخص لا تعرفه إطلاقاً

26) هل استشرت الطبيب أو السلطة المختصة رداً على ما حدث لك؟

نعم

لا، لا داعي لذلك

لا، لكنني أعتقد الآن أنه كان يجدر بي فعل ذلك

27) هل كنت في حالة سكر أو تخدير في المرة الأولى التي حدث ذلك في سياق أنشطة أو فعاليات ألعاب القوى؟

نعم

كلا

28) ما هي أشكال الإقناع أو الضغط أو القوة، التي استخدمها الشخص المعني، في ما له صلة بالأنشطة أو الفعاليات الرياضية؟

يمكن وضع علامة على عدة إجابات*.*

هل خدعك

أساء استخدام منصبه

أقنعك

هدّد بنبذك

حجزك

ضربك أو ألحق بك الأذى

أعطاك الكحول والمخدرات أو أقراص

أشكال أخرى

29) هل حاول الشخص المعني التعويض عليك في شكل هدايا أو أموال أو ما إلى ذلك؟

نعم

كلا

30) هل سبق لك أن طلبت المساعدة أو الدعم في الحالاات التالية:

نعم كلا

كونك ضحية للإساءة النفسية

كونك ضحية للإيذاء البدني

كونك ضحية للاعتداء الجنسي

الإبلاغ عن شخص ارتكب إساءة جنسية

مواجهة مشاكل مع الوالدين

المعاناة من مشاكل الصحة العقلية

حالات أخرى

31) الى من توجّهت للمساعدة؟

يمكن وضع علامة على عدة إجابات

الوالدان

الأخوة والأخوات

الصديق / الصديقة الحميم(ة)

صديق من العمر نفسه

أحد البالغين من العائلة أو صديق

"محترفون" - مدرّسون أو مستشارون أو دعم اجتماعي أو ممرضة أو ما شابه

"مسؤول ألعاب القوى" - مدرّب، أو مسؤول النادي، أو ما شابه

شخص آخر

أبلغت الخدمات الاجتماعية أو الشرطة

32) هل تلقيت الدعم والمساعدة التي احتجت إليهما؟

نعم

كلا

33) إذا كنت قد أبلغت عن حوادث مضايقة و/أو إساءة، فهل أنت راض عن الطريقة التي تم التعامل بها مع هذه الشكوى؟

نعم

كلا

34) هل أنت على علم بأي سياسة وقائية أو مدونة سلوك ينفّذها اتحادك الوطني؟

نعم

كلا

أرسل بياناتك عن طريق الضغط على زر **أرسل**.
